# A synopsis of knowledge, zoogeography and an online interactive map of Brazilian marine gastrotrichs

**DOI:** 10.7717/peerj.7898

**Published:** 2019-10-18

**Authors:** Ariane Campos, André Rinaldo Senna Garraffoni

**Affiliations:** Laboratory of Evolutionary Meiofauna, Department of Animal Biology, University of Campinas, Campinas, São Paulo, Brazil

**Keywords:** Gastrotricha, Chaetonotida, Biodiversity, Macrodasyida, Species distribution

## Abstract

Information regarding the records of Brazilian marine gastrotrichs is presented. We systematized and organized the existing information associated with approximately 23 species (belonging to the genera *Aspidiophorus*, *Chaetonotus*, *Crasiella*, *Dactylopodola*, *Dendrodasys*, *Draculiciteria*, *Halichaetonotus*, *Heteroxenotrichula*, *Kryptodasys*, *Macrodasys*, *Pseudostomella*, *Ptychostomella*, *Urodasys* and *Xenotrichula*) from the Brazilian coast (eight endemic) and their 1,581 records from 36 marine ecoregions of the world. A link is provided to an on-line interactive map where all occurrences for each species are shown, accompanied by geographic coordinates, oceans, countries, cities, granulometric characteristics and ecoregions. Furthermore, a critical analysis of the geographical distribution of Brazilian marine gastrotrichs, an estimate of the number of undescribed species, a summary of the existence and status of taxonomical collections are also presented.

## Introduction

Organism diversity is a result of historical processes, and all extant species are phylogenetically connected through time. Therefore, it is only possible to understand the structural changes that we observe in the light of evolution (Nunes & Christoffersen, 2009). Knowledge regarding biodiversity, history and interconnections is essential in understanding and anticipating the effects of disturbances, such as habitat destruction or the exchange of organisms between different localities, in the various systems (Schmidt-Rhaesa, 2002).

In the last decade, more than 20,000 marine species (9% of those currently known) have been described (Appeltans et al., 2012). The number of marine species described per year reached an all-time high in the past decade, with over 2,000 species described in each of four different years. The statistical model predicted a total of 540,000 marine species, with a 95% probability interval of 320,000 to 760,000. When stratified by the different taxonomic groups, the data were comparable to or less than the estimates. For taxonomic groups for which the majority of species remain to be described, the rate of discovery is still rising; therefore, the model could not make a meaningful estimate of total species numbers for some minor groups, which was the case for Gastrotricha (Appeltans et al., 2012).

Gastrotricha are microinvertebrates (from 60 to 3,500 μm in total body length) commonly found in marine and freshwater habitats that are recognized for their complex anatomy and life cycle, with a predominance of hermaphroditism (Ruppert, 1978; Weiss, 2001; Hummon, 2008; Guidi et al., 2014). Although gastrotrichs can be considered a cosmopolitan taxon found on all major continents (Kånneby & Hochberg, 2015), at least marine gastrotrichs show some level of endemism, mainly in the Northern Hemisphere (Garraffoni & Balsamo, 2017).

The taxon comprises approximately 860 species (Todaro, 2019a, 2019b), traditionally divided into the two orders: Macrodasyida Remane, 1925 (Rao & Clausen, 1970) and Chaetonotida Remane, 1925 (Rao & Clausen, 1970). Currently, the first order comprises 10 families, 36 genera and 377 described species (Todaro, 2019a), with strap-shaped bodies, the presence of pharyngeal pores and, usually, numerous adhesive tubes present in the anterior, lateral and posterior body regions (Todaro & Hummon, 2008; Kånneby & Hochberg, 2015). The majority of macrodasyids are marine species; only four species have been reported in freshwater from the Swiss Alps, Brazilian streams and a reservoir and aquifer from the USA (Ruttner-Kolisko, 1955; Kisielewski, 1987; Garraffoni et al., 2010, 2019a; Todaro et al., 2012; Araújo, Alcantara & Garraffoni, 2013; Kånneby & Wicksten, 2014; Kånneby & Kirk, 2017). Within Chaetonotida, eight families, 32 genera with nearly 483 species (Todaro, 2019b) are interstitial or epibenthic in marine and brackish water, and 2/3 of the species live in freshwater habitats (Balsamo et al., 2008, 2014). They are tenpin-shaped and have one pair (rarely two or any) of adhesive tubes, limited to the posterior end (Balsamo et al., 2008; Kånneby & Hochberg, 2015). The taxon Chaetonotida is divided into two suborders: Multitubulatina (monogeneric *Neodasys*) and Paucitubulatina. Within this order, the family Chaetonotidae is the most specious taxon, comprising approximately 1/3 of the species described for the whole group (Balsamo et al., 2014; Garraffoni & Balsamo, 2017).

The knowledge of South America marine gastrotrichs biodiversity was unknown (no species recorded) until a few decades ago (Hochberg, 2014). The first two mentions of the taxon in Brazil occurred when du Bois-Raymond (1952) reported an undescribed species of the genus *Thaumastoderma* collected at a three to five m depth off the coast of Ilhabela Island (Northern coast of São Paulo State) and Forneris (1985) cited an undescribed species of the genus *Macrodasys* sampled in the intertidal zone of Porchat Island (Santos region of São Paulo State). However, we can consider that the gastrotrich fauna were not discovered until the pioneer taxonomical study by Todaro & Rocha (2004) along the Northern coasts of the State of São Paulo (Ubatuba, Caragutatuba, Ilhabela and São Sebastião). In this first study, the authors described one new species, *Macrodasys fornerisae*, and reported 42 other species (most of these species were undescribed species belonging both Macrodasyida and Chaetonotida). One year later, Todaro & Rocha (2005) reported results of a second study, mostly conducted on the Northern coasts of the State of São Paulo and, for the first time, in the southern part of the State of Rio de Janeiro (Paraty). In this study, the authors found 30 species, most of which had also been found in the first study, but they also reported some species not formally described and species recorded for the first time in Brazil. Subsequently, Todaro (2012, 2013) described the new species *Pseudostomella dolichopoda* and *Ptychostomella lamelliphora*, that were already reported as *Pseudostomella* sp. and *Ptychostomella* sp., respectively, in Todaro & Rocha (2004, 2005). All these studies were part of a larger research program studying the diversity of marine invertebrates of the Northern coasts of the State of São Paulo (Migotto & Tiago, 1999).

During the workshop “Taxonomy and Diversity of Marine Meiofauna” held in São Sebastião, State of São Paulo (Fonseca, Norenburg & Di Domenico, 2014), Hochberg (2014) described the new species *Crasiella fonseci*. Additionally, Araújo, Balsamo & Garraffoni (2014) described another species belonging to the genus *Pseudostomella* (*Pseudostomella squamalongispinosa*). These authors found and described a new species of marine Gastrotricha from Brazil outside of the State of São Paulo; the new species was found on the Southern coast of the State of Bahia (Nova Viçosa).

Araújo et al. (2016) reported a new record of *Pseudostomella dolichopoda* Todaro, 2012, originally described from the Northern coast of São Paulo, and then collected from the State of Espírito Santo. Garraffoni, Di Domenico & Amaral (2016) analyzed the patterns of diversity of marine Gastrotricha among benthic habitats and localities along the Southeastern Brazilian coast. The authors concluded that the diversity patterns of Brazilian marine gastrotrichs could be explained by differences in sediment textures, tidal zones, and localities. Garraffoni, Di Domenico & Hochberg (2017) reported new records of marine gastrotrichs from sublittoral sediments around São Sebastião Island (where the municipality of Ilhabela is housed). Species belonging to the genus *Acanthodasys* (Macrodasyida) were reported for the first time in the Southern Hemisphere, and *Dactylopodola todaroi* was described as a new species. Recently, Todaro et al. (2019) described a new Macrodasyida genus (*Kryptodasys*) with three distinct species from Italy, Sweden and Brazil. The Brazilian species (*Kryptodasys carlosrochai*) appeared as “*nov. gen. nov. spec*.” in Todaro & Rocha (2004).

The aim of the present study was to systematize and organize the knowledge about the marine Gastrotricha diversity on the Brazilian coast, with a species inventory, critical analysis of geographical distribution patterns of these species and some future perspectives about the study of these taxa in Brazil.

## Materials and Methods

### Marine ecoregions of the world

We avoided using geopolitical boundaries to analyze gastrotrich distributions because the delimitation of such areas does not reflect natural units (Nihei, 2006). Thus, we used the biogeographic regionalization for coastal and shelf areas proposed by Spalding et al. (2007) to establish the distribution patterns of species. The hierarchical system proposed by these authors has three levels of inclusiveness: ecoregion (smallest-scale unit), province (nested within the realm) and realm (largest spatial unit). In the present study, we used the spatial unit ecoregion, as it is defined as “Areas of relatively homogeneous species composition, clearly distinct from adjacent systems. The species composition is likely to be determined by the predominance of a small number of ecosystems and/or a distinct suite of oceanographic or topographic features” (Spalding et al., 2007).

The system proposed by Spalding et al. (2007) is composed of 232 ecoregions covering all coastal and shelf waters of the world. Among them, five ecoregions occur in Brazil: Amazônia, Northeastern Brazil, Eastern Brazil, Southeastern Brazil and Rio Grande.

**Figure 1:**
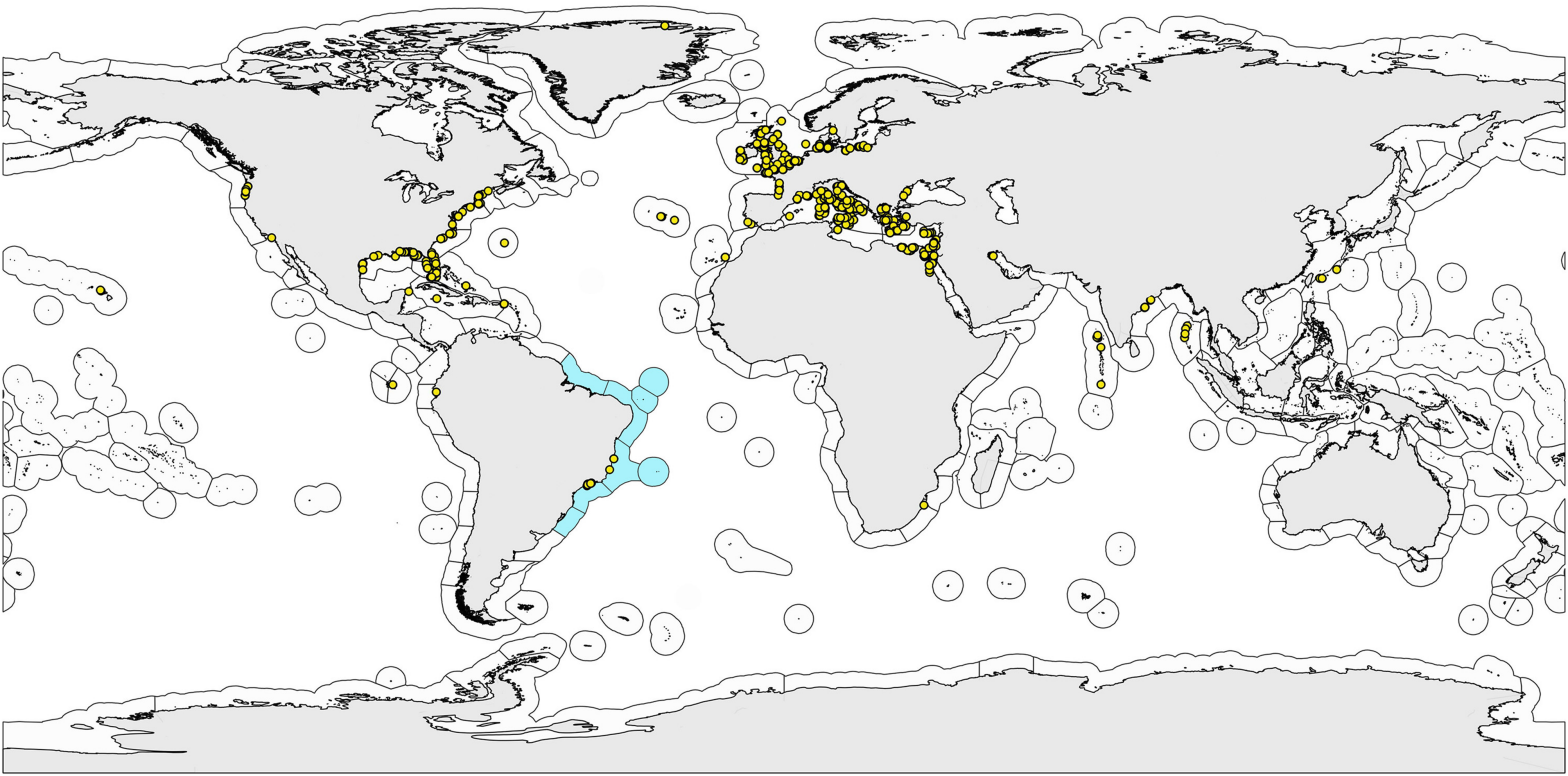
World map with the bioregionalization hierarchical system of ecoregions. World map with the bioregionalization hierarchical system of ecoregions according to Spalding et al. (2007). Ecoregions observed on the Brazilian coast are colored light blue. Dots are the sampling sites of the marine gastrotrich species found in Brazil (endemic or not).

The distribution map (Fig. 1) with the known records of Brazilian Gastrotricha species and the worldwide ecoregions was made with the software Quantum GIS (http://www.qgis.org).

### Literature database

Distribution data on marine gastrotrichs up to 2010 were obtained from the “Global distribution of marine Gastrotricha” compilation by Dr. William D. Hummon (Todaro, 2017), and from 2011 to 2019, we gathered data directly from the literature (see complete list in Garraffoni & Balsamo, 2017; Todaro et al., 2019) (Fig. 1; Data S1).

### Brazilian species records

The species lists of Brazilian gastrotrichs followed the classification proposed by Todaro (2019a, 2019b).

Below the name of each species there is mention about the type locality, a summary of records per country, and the total number of records in Brazil and the world. When possible, a brief remark about the current species distribution and taxonomy status is noted. The list of the geographic coordinates of the locality sites where each species was found are arranged following the world bioregionalization framework of ecoregions (the ecoregions number are the same as those reported by Spalding et al. (2007)), countries and beaches.

### Interactive map

All species listed in this paper were entered into a spreadsheet, and an interactive map was produced using My Maps in Google Drive. The map can be accessed at https://www.arcgis.com/apps/View/index.html?appid=4018bc0c77644422a761b8c213eb9c73.

**Figure 2:**
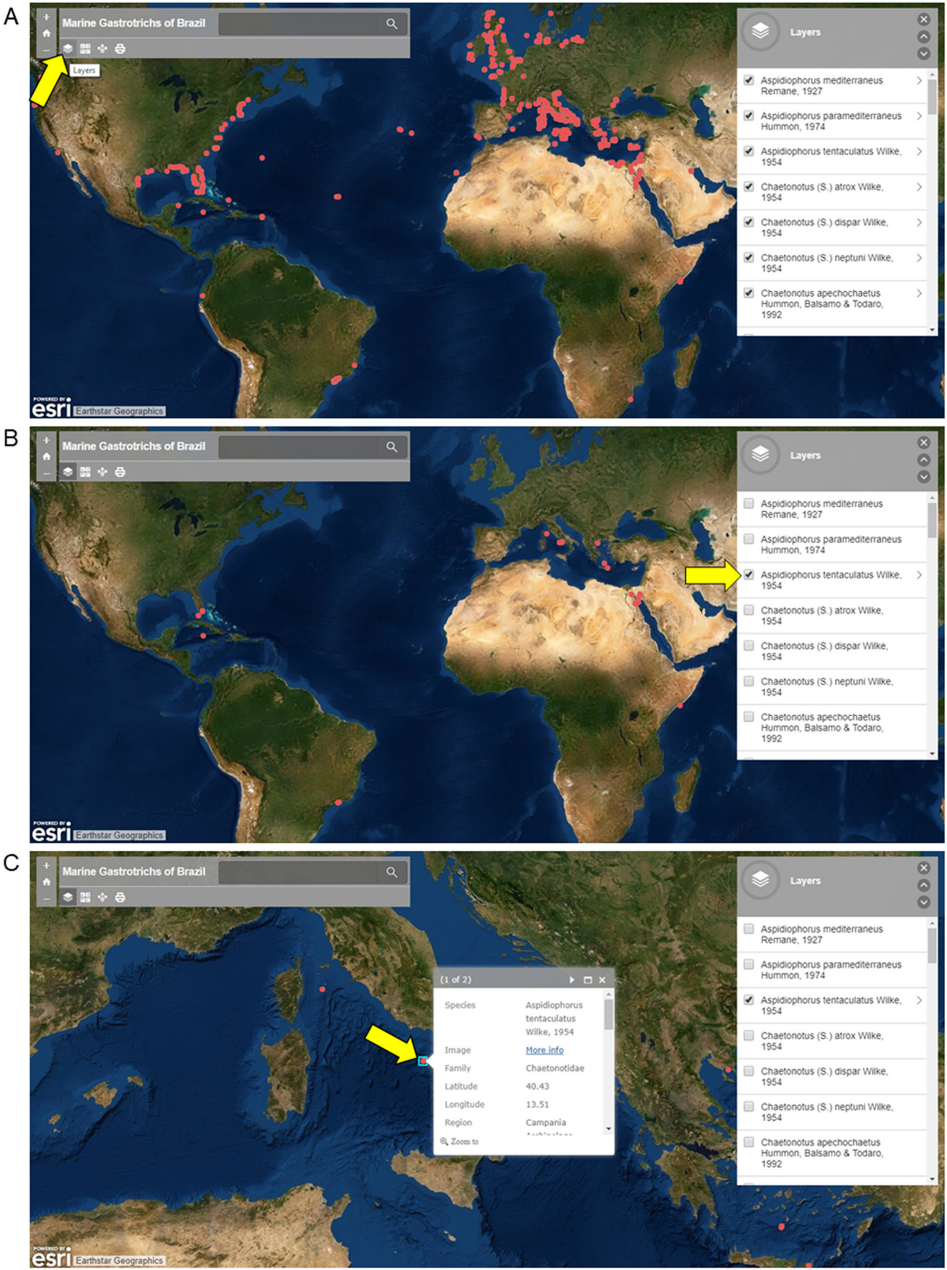
Screenshots of the interactive map of Marine Gastrotrichs of Brazil. Screenshots of the interactive map of Marine Gastrotrichs of Brazil. (A) All records available. (B) *Aspidiophorus tentaculatus* records. (C) Image and select data of a specific record of *Aspidiophorus tentaculatus*.

Screenshots from the ArcGis, 2019 Online platform of Marine Gastrotrichs of Brazil are shown in Fig. 2 for instructional purposes. Three views are available: (A) Map view with all records available (B) Map view with only specific species records (C) Map view with image and select data a specific record. Names of species can be displayed using the layer function, where species occurrences can be filtered (Figs. 2A and 2B). Each occurrence in the map is clickable, resulting in a window showing an image of the species and information about the record (Fig. 2C).

Schematic drawings of those species formally described in Brazil were redrawn from original descriptions or redescriptions. Micrographs of *Xenodasys* sp. were used to exemplify the species collected by researchers from the Laboratory of the Meiofaunal Organisms Evolution and yet not formally described. In this case, samples of the upper sediment layer were collected with a manual corer, and in the laboratory, the specimens were sorted under a stereomicroscope Zeiss DM2000, mounted on glass slides, observed in vivo under a Zeiss Axioskop 2 plus equipped with differential interference contrast and AxioCam MRC5 digital video camera.

#### Permits

This study was approved by SISBIO (Ministério do Meio Ambiente—project number: 27654-1).

## Results

To date, specimens of 23 species were collected from the Brazilian coast, and all of these named taxa (at the species level) are considered valid according to modern standards (Table 1). The order Chaetonotida is the richest in species with 14 species (Chaetonotidae Zelinka, 1889: *Aspidiophorus mediterraneus* Remane, 1927; *A. paramediterraneus* Hummon, 1974; *A. tentaculatus* Wilke, 1954; *Chaetonotus* (*C*.) *apechochaetus* Hummon, Balsamo et Todaro, 1992; *Chaetonotus* (*S*.) *atrox* Wilke, 1954; *Chaetonotus* (*S*.) *dispar* Wilke, 1954; *Chaetonotus* (*S*.) *neptuni* Wilke, 1954; *Halichaetonotus decipiens* (Remane, 1929); *Halichaetonotus marivagus* Balsamo, Todaro et Tongiorgi, 1992; *Halichaetonotus euromarinus* Hummon et Todaro 2010; Xenotrichulidae Remane, 1927: *Draculiciteria tesselata* (Renaud-Mornant, 1968); *Heteroxenotrichula pygmaea* (Remane, 1934); *Heteroxenotrichula squamosa* Wilke, 1954; *Xenotrichula intermedia* Remane, 1934. In contrast, nine species are listed within the order Macrodasyida (Thaumastodermatidae Remane, 1927: *Pseudostomella dolichopoda* Todaro, 2012, *Pseudostomella squamalongispinosa* Araújo, Balsamo et Garraffoni, 2014, *Ptychostomella lamelliphora* Todaro, 2013; Planodasyidae Rao et Clausen, 1970: *Crasiella fonseci* Hochberg, 2014; Macrodasyidae Remane, 1926: *M. fornerisae* Todaro et Rocha, 2004, *Urodasys viviparus* Wilke, 1954, *K. carlosrochai* Todaro, Dal Zotto, Kånneby, Hochberg, 2019; Dactylopodolidae Strand, 1929: *Dactylopodola baltica* (Remane, 1926), *Dactylopodola todaroi* Garraffoni, Di Domenico et Hochberg, 2017).

**Table 1:** Geographic distribution and species list of georeferenced marine gastrotrichs records in Brazil and other localities in different ecoregions reported by Spalding et al. (2007).

Eco name	Eco n°	List of species	N° of taxa	N° of genera
Northern Norway and Finnmark	23	*Het. intermedia*	1	1
Baltic Sea	24	*Het. intermedia*, *Dac. baltica*	2	2
North Sea	25	*Asp. mediterraneus*, *Asp. paramediterraneus*, *Cha. atrox*, *Cha. neptuni*, *Hal. decipiens*, *Hal. euromarinus*, *Het. pygmaea*, *Het. squamosa*, *Het. intermedia*, *Dra. tesselata*, *Dac. baltica*	11	6
Celtic Seas	26	*Asp. mediterraneus*, *Asp. paramediterraneus*, *Cha. atrox*, *Cha. dispar*, *Cha. neptuni*, *Hal. decipiens*, *Hal. euromarinus*, *Het. pygmaea*, *Het. squamosa*, *Het. intermedia*, *Dra. tesselata*, *Dac. baltica*	12	6
South European Atlantic Shelf	27	*Asp. mediterraneus*, *Asp. paramediterraneus*, *Hal. decipiens, Hal. euromarinus*, *Het. squamosa*, *Het. intermedia*, *Dra. tesselata*	7	4
Azores Canaries Madeira	29	*Cha. atrox*, *Het. pygmaea*, *Dra. tesselata*	3	3
Adriatic Sea	30	*Asp. mediterraneus*, *Asp. paramediterraneus*, *Cha. apechochaetus*, *Cha. atrox*, *Cha. dispar*, *Cha. neptuni*, *Hal. decipiens*, *Hal. euromarinus*, *Het. pygmaea*, *Het. squamosa*, *Het. intermedia*, *Dra. tesselata*, *Uro. viviparus*	13	6
Aegean Sea	31	*Asp. mediterraneus*, *Asp. paramediterraneus; Asp. tentaculatus*, *Cha. apechochaetus*, *Cha. atrox*, *Cha. neptuni*, *Hal. decipiens*, *Hal. euromarinus*, *Hal marivagus, Het. pygmaea, Het. squamosa, Het. intermedia, Dac. baltica, Uro. viviparus*	14	6
Levantine Sea	32	*Asp. paramediterraneus, Cha. apechochaetus, Cha. atrox, Cha. neptuni, Hal. decipiens, Hal. euromarinus, Het. pygmaea, Het. squamosa, Het. intermedia, Dac. baltica, Uro. viviparus*	11	6
Ionian Sea	34	*Asp. mediterraneus, Asp. paramediterraneus, Cha. apechochaetus, Cha. atrox, Cha. dispar, Cha. neptuni, Het. squamosa, Het. intermedia, Dra. tesselata, Uro. viviparus*	10	5
Western Mediterranean	35	*Asp. mediterraneus, Asp. paramediterraneus, Asp. tentaculatus, Cha. apechochaetus, Cha. atrox, Cha. dispar, Cha. neptuni, Hal. decipiens, Hal. euromarinus, Hal marivagus, Het. pygmaea, Het. squamosa, Het. intermedia, Dra. tesselata, Uro. viviparus*	15	6
Gulf of Maine/Bay of Fundy	40	*Asp. mediterraneus, Asp. paramediterraneus, Hal. decipiens, Hal. euromarinus, Het. pygmaea, Het. squamosa, Het. intermedia, Dra. tesselata, Dac. baltica*	9	5
Virginian	41	*Asp. mediterraneus, Asp. paramediterraneus, Cha. atrox, Cha. dispar*, *Hal. euromarinus*, *Het. pygmaea*, *Het. squamosa*, *Het. intermedia*, *Dac. baltica*	9	5
Carolinian	42	*Asp. mediterraneus*, *Asp. paramediterraneus*, *Cha. neptuni*, *Hal. euromarinus*, *Het. pygmaea*, *Het. squamosa*, *Het. intermedia*, *Dra. tesselata*, *Dac. baltica*	9	6
Northern Gulf of Mexico	43	*Asp. mediterraneus*, *Asp. paramediterraneus*, *Cha. atrox*, *Cha. dispar*, *Hal. decipiens*, *Hal. euromarinus*, *Het. pygmaea*, *Het. squamosa*, *Het. intermedia*, *Dra. tesselata*	10	5
Black Sea	44	*Asp. mediterraneus*, *Hal. decipiens*, *Het. pygmaea*, *Het. intermedia*	4	3
Oregon, Washington, Vancouver Coast and Shelf	57	*Het. pygmaea*	1	1
Southern California Bight	59	*Het. intermedia*	1	1
Bermuda	62	*Uro. viviparus*	1	1
Bahamian	63	*Asp. mediterraneus*, *Asp. paramediterraneus*, *Het. pygmaea*	3	2
Eastern Caribbean	64	*Asp. paramediterraneus*, *Cha. dispar*, *Het. pygmaea*, *Dra. tesselata*	4	4
Southwestern Caribbean	67	*Uro. viviparus*	1	1
Greater Antilles	65	*Asp. tentaculatus*	1	1
Western Caribbean	68	*Asp. paramediterraneus*	1	1
Floridian	70	*Asp. mediterraneus*, *Asp. paramediterraneus*, *Asp. tentaculatus*, *Cha. atrox*, *Cha. dispar*, *Cha. neptuni*, *Hal. euromarinus*, *Het. pygmaea*, *Het. squamosa*, *Dra. tesselata*, *Dac. baltica*, *Uro. viviparus*	12	7
Eastern Brazil	76	*Pse. dolichopoda*, *Pse. squamalongispinosa*	2	1
Northern and Central Red Sea	87	*Asp. mediterraneus*, *Asp. paramediterraneus*, *Asp. tentaculatus*, *Cha. apechochaetus*, *Cha. atrox*, *Cha. neptuni*, *Hal. decipiens*, *Het. pygmaea*, *Het. squamosa*, *Uro. viviparus*	10	5
Central Somali Coast	93	*Asp. mediterraneus*, *Asp. paramediterraneus*	2	1
Maldives	105	*Cha. atrox*, *Uro. viviparus*	2	2
Eastern India	107	*Cha. atrox*, *Het. intermedia*, *Uro. viviparus*	3	2
Andaman and Nicobar Islands	109	*Cha. atrox*, *Uro. viviparus*	2	2
South Kuroshio	121	*Cha. atrox*, *Het. pygmaea*	2	2
Hawaii	153	*Asp. paramediterraneus*, *Het. pygmaea*	2	2
Guayaquil	171	*Asp. paramediterraneus*, *Het. intermedia*	2	2
Eastern Galapagos Islands	173	*Het. pygmaea*	1	1
Southeastern Brazil	180	*Asp. mediterraneus*, *Asp. paramediterraneus*, *Asp. tentaculatus*, *Cha. apechochaetus*, *Cha. atrox*, *Cha. dispar*, *Cha. neptuni*, *Hal. decipiens*, *Hal. euromarinus*, *Hal marivagus*, *Het. pygmaea*, *Het. squamosa*, *Het. intermedia*, *Dra. tesselata*, *Cra. fonseci*, *Dac. baltica*, *Dac. todaroi*, *Den*. aff. *rubomarinus*, *Mac. fornerise*, *Uro. viviparus*, *Pse. dolichopoda*, *Pse. squamalongispinosa*, *Pty. lamelliphora*	23	12

**Note:**

Eco, Ecoregion; N, Number; *Asp., Aspidiophorus*; *Cha., Chaetonotus*; *Hal., Halichaetonotus*; *Het., Heteroxenotrichula*; *Dra., Draculiciteria*; *Cra., Crasiella*; *Dac., Dactylopodola*; *Den., Dendrodasys*; *Mac., Macrodasys*; *Uro., Urodasys*; *Pse., Pseudostomella*; *Pty., Ptychostomella*.

Only seven species (30%), all belonging to the order Macrodasyida (*Pseudostomella dolichopoda*, *Pseudostomella squamalongispinosa*, *Ptychostomella lamelliphora*, *Crasiella fonseci*, *M. fornerisae*, *Dactylopodola todaroi*, *K. carlosrochai*), were originally described from samples collected in Brazil (Table 1). Since the first marine gastrotrich described in Brazil were published in 2004 and the last one in 2019 (Araújo, Balsamo & Garraffoni, 2014; Araújo et al., 2016; Garraffoni, Di Domenico & Hochberg, 2017; Hochberg, 2014; Todaro, 2012, 2013; Todaro & Rocha, 2004, 2005; Todaro et al., 2019) the historical rates of species description are 0.43/per year, much lower when compared, in the same period, with the world historical rates of marine gastrotrich descriptions (8.93/per year).

**Figure 3:**
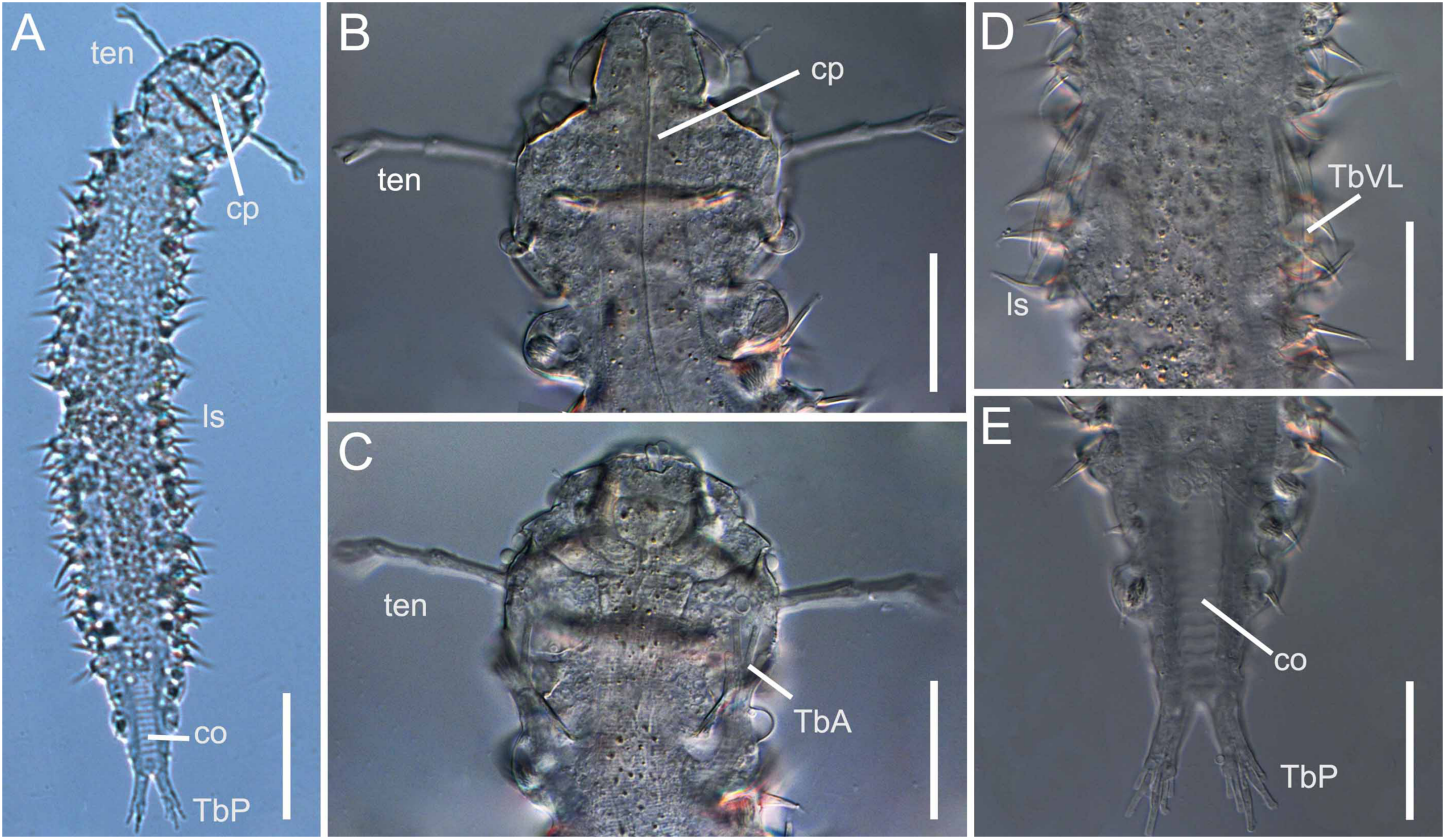
Xenodasys sp., DIC images from Brazil (A) Habitus. (B) Dorsal view of the head region with tentacles. (C) Ventral view of the head region with tentacles. (D) Ventral view of the middle body with ventrolateral adhesive tubes. (E) Ventral view of the posterior end. Abbreviations: co, chordoidorgan; cp, cephalic plates; lsp, lateral spines; TbA, anterior adhesive tubes; TbP, posterior adhesive tubes; TbVL, ventrolateral adhesive tubes; ten, head tentacles. Scales: (A) 45 μm; (B–E) 20 μm.

The number of species recorded from the Brazilian coast was only 25% of the total marine gastrotrich richness estimate, as more than 40 species were collected but not yet formally described. Some of these unpublished species, for example, *Xenodasys* sp. (Fig. 3) (Xenodasyidae), *Acanthodasys* sp. 1 and sp. 2 (Thaumastodermatidae—Garraffoni, Di Domenico & Hochberg, 2017), *Mesodasys* sp., *Dolichodasys* sp. (Cephalodasyidae—Todaro & Rocha, 2004, 2005), *Dendrodasys* sp. (Dactylopodolidae—Todaro & Rocha, 2004), *Dendrodasys* aff. *rubomarinus* (Dactylopodolidae—Garraffoni, Di Domenico & Hochberg, 2017) are mentioned for the first time in the Southern Hemisphere.

Gastrotrichs samplings were performed on at least 38 distinct beaches along the Brazilian coastline: 26 in the State of São Paulo (69%), five in the State of Rio de Janeiro (13%), three in the States of Bahia (7%) and Paraná (7%) and one in the State of Espírito Santo (4%).

Records of Brazilian marine nominal species were reported from 37 ecoregions, covering 6.2% of the 232 marine ecoregions in the world (Table 1). Among the nine ecoregions of the Brazilian coast, only two (22%) registered the occurrence of species, but a strong bias was observed; one of them, Southeastern Brazil (which encompasses the Northern coast of São Paulo State and the Southern coast of Rio de Janeiro), contained 97% of the sampling sites in Brazil (Table 1). Furthermore, the gastrotrich species recorded to have a very heterogeneous geographic distribution, with few restricted/endemic species (27%) and species with a relatively wide distribution, such as *Heteroxenotrichula pygmaea* and *A. paramediterraneus* present in 21 ecoregions, *Heteroxenotrichula intermedia* in 19 ecoregions, *A. mediterraneus* and *Chaetonotus* (*S*.) *atrox* in 17 ecoregions and *Halichaetonotus euromarinus*, *Draculiciteria tesselata* and *U. viviparus* in 13 ecoregions (Table 1).

## Discussion

### Historical study of Brazilian Gastrotricha

This is the first historical review on the gastrotrichs occurring along the Brazilian coast and the first synthesis of the available literature shown as an annotate species checklist. Although the obtained data showed scattered references from Brazilian literature, this panorama has started to change slowly. Historically, in Brazil (and in many other countries of Southern Hemisphere, Hummon, 1974; Hochberg, 2003, 2008; Todaro, Perissinotto & Bownes, 2015; Todaro, Dal Zotto & Leasi, 2015; Todaro et al., 2017), only sporadic collections were carried out by foreign researchers, and the involvement of Brazilian researchers could thus be considered incipient (Balsamo et al., 2014; Garraffoni & Balsamo, 2017). However, a few years ago, the senior author of the present study became the first active native researcher and started to coordinate a research group interested in uncovering the systematics, evolution and biogeography of gastrotrichs. This group has grown over time allowing to start several research projects on these topics.

Garraffoni (2017) noted three major gaps in the current knowledge about Brazilian freshwater Gastrotricha: (a) most of the Brazilian inland waters had never been sampled before. Records, in fact, show a strong bias because most of the samplings were conducted in the State of São Paulo, and only a few in other states); (b) identification keys for a great number of genera do not exist and there is difficulty in obtaining classic and old literature from the end of the century XIX and early century XX; and (c) reference collections about the taxon in Brazilian Museums are absent (see Garraffoni et al., 2019b).

Unfortunately, most of these gaps are also observed for marine animals. Most of the surveys were concentrated on the Northern coast of São Paulo State and the southern coast of Rio de Janeiro State (Todaro & Rocha, 2004, 2005; Todaro, 2012, 2013; Hochberg, 2014; Garraffoni, Di Domenico & Hochberg, 2017; Todaro et al., 2019), and only two studies were performed outside this area (Araújo, Balsamo & Garraffoni, 2014: Bahia State; Araújo et al., 2016: Espírito Santo State) (see below section “Brazilian Gastrotricha distribution patterns”).

The unique Brazilian collection, with adequate and permanent curatorial attention in Brazil, which contains type material regarding marine Gastrotricha is located in the Zoological Museum “Adão José Cardoso” (ZUEC) of the State University of Campinas. This museum houses the type series for *Pseudostomella squamalongispinosa* Araújo, Balsamo et Garraffoni 2014 (GCH 02-04), *Dactylopodola todaroi* Garraffoni, Di Domenico et Hochberg, 2016 (GCH 26-28) and *Crasiella fonseci* Hochberg, 2014. Regarding this last species, due to problems in sending back the type material to Brazil (R. Hochberg, 2018, personal communication), in the original description, it was only mentioned that the holotype was deposited at the ZUEC, but without an accession number. However, we had the possibility to obtain the original type material and deposit it at the ZUEC as GCH-51. Furthermore, additional material was deposited for *Pseudostomella dolichopoda* Todaro, 2012 (GCH 29). However, the representation of the Gastrotricha physical specimens in Brazilian collections (and also in the world) is extremely poor which is partially reasoned by the gastrotrichs specifies (see Garraffoni et al., 2019b).

The only significant difference between Brazilian marine and freshwater gastrotrichs is related to the number of available identification keys; that mainly exists for marine species. In the last years, taxonomic keys for species belonging to six genera were published, and many listed taxa in Brazil, for example, *Aspidiophorus* (Todaro et al., 2009); *Cephalodasys* (Kieneke, Schmidt-Rhaesa & Hochberg, 2015); *Dactylopodola* (Von Und Zu Gilsa et al., 2014); *Paraturbanella* (Todaro et al., 2017); *Pseudostomella* (Clausen, 2004; Todaro, 2012; Araújo et al., 2016) and *Tetranchyroderma* (Todaro, 2002).

If the taxonomic knowledge of Brazilian gastrotrichs is far from appropriate, also studies on other biological aspects, such as ecology or natural history, are totally absent.

### Brazilian Gastrotricha distribution patterns

In general, in recent years, the number of new Gastrotricha species descriptions increased in geographic locations known for poor sampling (Todaro & Rocha, 2004, 2005; Hochberg, 2003, 2008, 2014; Todaro, 2012, 2013; Hochberg, Atherton & Gross, 2013; Von Und Zu Gilsa et al., 2014; Araújo, Balsamo & Garraffoni, 2014; Araújo et al., 2016; Todaro, Leasi & Hochberg, 2014; Todaro, Perissinotto & Bownes, 2015; Todaro, Dal Zotto & Leasi, 2015; Todaro et al., 2017, 2019; Kieneke, Schmidt-Rhaesa & Hochberg, 2015; Garraffoni, Di Domenico & Amaral, 2016; Garraffoni, Di Domenico & Hochberg, 2017; Araújo & Hochberg, 2017; Chatterjee, Priyalakshmi & Todaro, 2019).

In this scenario, when compared the 12 countries of South America, only four (Colombia: Hummon, 1974; Ecuador, Galapagos Island: Schmidt, 1974; and Uruguay: Dioni, 1960) have information available about Gastrotricha fauna, and Brazil can be considered the country with more studies and and descriptions. However, when only Brazil, with a coastline encompassing almost 7,500 km and varying between ~5° and ~25° of longitude, was analyzed, it was found that more than 95% of the country has never been never sampled. However, most of the scientific effort was placed only in the cost of São Paulo State (Todaro & Rocha, 2004, 2005; Todaro, 2012, 2013; Hochberg, 2014; Garraffoni, Di Domenico & Amaral, 2016; Garraffoni, Di Domenico & Hochberg, 2017; Todaro et al., 2019; Supplemental Material 1). It is important to highlight that these results in the State of São Paulo were not accidental but due to many initiatives for funding studies aimed to reveal the biodiversity of fauna and flora in the State that were supported by the Virtual Institute of Biodiversity, BIOTA-FAPESP, organized by FAPESP, the State of São Paulo Research Foundation (Migotto & Tiago, 1999; Joly et al., 2010).

However, if we compared the number of sampled beaches along the São Paulo coastline (26) with the total number of beaches in continental and islands in the State (342), the station coverage is very low, with a very limited number of sampled sites (7% of the total beaches in the state). If considered the states of São Paulo and Rio de Janeiro, which together host 85% of the sampled sites located in Brazil, the sampling site is even worse (4% of the total beaches in both states). For comparison, Italy has a long-standing tradition in the study of Gastrotricha (Balsamo et al., 2014) and the Italian coastline encompasses almost 7,800 km. In this country, gastrotrichs were sampled in 246 localities and comprise 177 different species (Todaro et al., 2003).

As noted above, this difference regarding the knowledge of gastrotrich biodiversity between the Northern and Southern hemispheres occurs because the first studies dedicated to this group of animals were conducted by European or North-American Institutions (Balsamo et al., 2014; Garraffoni & Balsamo, 2017). Unfortunately, many other meiofauna taxa (organisms that pass through 500 μm mesh, but is retained by 44 μm one) present this bias, that is, the distribution of taxon seems to reflect the distribution of specialist more than that of taxon itselves. This bias is commonly known as “rotiferologist” effect (Fontaneto et al., 2012). Thus, this discrepancy in information between the two hemispheres has some consequences; (a) the first record and description of a marine gastrotrichs in the Mediterranean was done by Claparède (1867). In contrast, the first mention of a Brazilian gastrotrich was noted by Eveline du Bois-Raymond Marcus 85 years after the René-Édouard Claparède publication (du Bois-Raymond, 1952), and it was approximately 140 years before Antonio Todaro and Carlos Rocha named the first new species from Brazil (Todaro & Rocha, 2004); (b) 70% of the marine gastrotrich sampling sites around the world are located in the Northern Hemisphere (mainly in Atlantic coast of the United States, German, Poland, Mediterranean and Great Britain coasts) (Garraffoni & Balsamo, 2017); (c) In recent years, the number of studies dealing with the reconstruction of intraphylum relationships of Gastrotricha based on molecular data has increased (Todaro et al., 2011, 2012; Todaro, Leasi & Hochberg, 2014; Todaro, Dal Zotto & Leasi, 2015). However, the DNA used in these studies was mainly extracted from species collected in the Northern Hemisphere.

Despite the bias in sampling sites distribution, the geographic distribution of the Brazilian marine gastrotrichs possesses relatively restricted areas, that is, one or two ecoregions (six species) to widespread distributions, that is, at least four ecoregions (22 species). These numbers have a totally opposite tendency compared to marine gastrotrichs worldwide, as 80% of the species are distributed in only two ecoregions (A.R.S. Garraffoni et al., unpublished data). As a consequence, the absence of wide-scale reviews in the country can produce uncontextualized taxonomic revisions and spurious data on biodiversity. Finally, in recent years, the ubiquitous distribution of marine gastrotrichs has been challenged (Curini-Galletti et al., 2012; Kieneke, Martinez-Arbizu & Fontaneto, 2012; Garraffoni, Di Domenico & Amaral, 2016; Garraffoni & Balsamo, 2017), and the number of widespread species appears not as high as previously thought.

### Brazilian Gastrotricha richness

As reported above, due to the low number of taxonomic studies and sample bias along the Brazilian coast, we can provide the estimated richness of gastrotrichs only for the coasts of the states of São Paulo and Rio de Janeiro (Garraffoni, Di Domenico & Amaral, 2016). In this region, besides the 23 formally described species, at least 40 not formally described species were recorded (Todaro & Rocha, 2004, 2005; Garraffoni, Di Domenico & Amaral, 2016; Garraffoni, Di Domenico & Hochberg, 2017): thus, we can say that at least 20 species of marine gastrotrichs from only 5% of the Brazilian coast are waiting to be described. Furthermore, many of these undescribed species belong to very rare taxa, for example, *Diplodasys* sp., *Dolichodasys* sp., *Mesodasys* sp., *Paradasys* sp., (Todaro & Rocha, 2004), sometimes never reported before from the Southern Hemisphere, for example, *Acanthodasys* sp1 and sp2 (Garraffoni, Di Domenico & Hochberg, 2017) or *Xenodasys* sp. collected by the authors of the present study in December 2018 at Fome Beach, Ilhabela Island, Northern coast of São Paulo State (Fig. 3). The last species belongs to a genus with only three species, each with regional (limited) distributions (Schuster et al., 2018).

The large number of unknown species found on the southeastern Brazilian coast is not a surprise. To date, 507 marine gastrotrichs (Garraffoni & Balsamo, 2017; Todaro, 2019a) have been described from all the world’s oceans, but at least 2,244–3,244 species are still unknown and undiscovered (Appeltans et al., 2012). Using species richness as a metric for assessment of the worldwide marine gastrotrich biodiversity, only less than 20% are known (Appeltans et al., 2012).

## Conclusions

Although the biogeographical knowledge about Brazilian gastrotrich fauna is greatly hampered by regional discrepancies in taxonomic knowledge, there is no doubt about the high diversity of the group and that many new species will be described. Thus, to achieve a fairly realistic number of Gastrotricha species in Brazil, we need to increase the number of species inventories carried out outside of the coasts of the states of São Paulo and Rio de Janeiro. This initiative will be only possible stimulating the establishment of network of researchers in order to contribute to increase regional scientific initiative. The on-line map can be interpreted as a starting point to increase the understanding of the diversity and biogeographic patterns of gastrotrichs in Brazil and their affinities with other geographic regions.

## Supplemental Information

10.7717/peerj.7898/supp-1 Supplemental Information 1 List of marine gastrotrichs species (formally described) records throughout Brazilian coast and the world. Wo, world (except Brazil); BR, Brazil.

## Additional Information and Declarations

### Field Study Permissions

The following information was supplied relating to field study approvals (i.e., approving body and any reference numbers): Field trips were aproved by SISBIO (Ministério do Meio AMbiente) under project number: 27654-1.

### Data Availability

The following information was supplied regarding data availability: The raw geographic coordinates and some biogeographic issues are available in the Supplemental File. The raw data shows countries and beaches where the species were found. The interactive map is available at ArcGIS: https://www.arcgis.com/apps/View/index.html?appid=4018bc0c77644422a761b8c213eb9c73.
