# Supplementary material for: A synopsis of knowledge, zoogeography and an online interactive map of Brazilian marine gastrotrichs
Source: PeerJ. 2019 Oct 18;7:e7898. doi: 10.7717/peerj.7898 (PMC6802584; doi:10.7717/peerj.7898)
Supplement: Supplemental Information 1 — Wo, world (except Brazil); BR, Brazil. [file peerj-07-7898-s001.docx]

**Synopsis of the knowledge, zoogeography and an on-line interactive map** **of Brazilian marine gastrotrichs**

Ariane Campos and André Rinaldo Senna Garraffoni

List of marine gastrotrichs species (formally described) records throughout Brazilian coast and the world. Wo: world (except Brazil), BR: Brazil

**Order** Chaetonotida Remane, 1925 (Rao and Clausen, 1970)

**Family** Chaetonotidae Zelinka, 1889

**Genus** *Aspidiophorus* Voigt, 1903

*Aspidiophorus mediterraneus* Remane, 1927

Terra typica: Mergellina, Campania, Italy: 40°,50'N/14°,14'E

Record numbers: DENMARK: (1); GERMANY: (3); SCOTLAND:(4); IRELAND: (1); CHANNEL ISLES:(2); FRANCE: (8); PORTUGAL: (1); ITALY: (44); GREECE: (5); UNITED STATES OF AMERICA: (25); BULGARIA: (1); ROMANIA: (3); EGYPT: (9); ISRAEL: (1); SOMALIA: (1); BRAZIL: (3); total: Wo: (109); Br: (3)

25. North Sea - DENMARK: Romo: 55°,04'N/08°,33'E; GERMANY: Deutche Bucht: 54°,13'N/08°,00'E; Litoral Station: 55°,01'N/08°,26'E; Puan Klent: 54°,48'N/08°,18'E; SCOTLAND: Rattray: 57°,34'N/01°,50'W.

26. Celtic Seas - IRELAND: Derrymore Strand: 52°,13'N/09°,51'W; CHANNEL ISLES: Corbiere Point: 49°,10'N/02°,16'W; Vazon Bay: 49°,28'N/02°,36'W; SCOTLAND: Ballantrae: 55°,06'N/05°,00'W; Irvine Bay A: 55°,37'N/04°,44'W; Irvine Bay C: 55°,36'N/04°,42'W; FRANCE: Ile de Batz: 48°,44'N/04°,00'W; Banc de Duslen/Pighet: 48°,43'N/03°,58'W; Courseules: 49°,18'N/00°,28'W; Luc-sur-Mer: 49°,16'N/00°,21'W; Lagune du Brusc: 43°,03'N/05°,47'E; Goudes: 43°,12'N/05°,20'E; Pointe Rouge: 43°,13'N/05°,21'E; Plage d. Sablettes: 43°,03'N/05°,59'E

27. South European Atlantic Shelf - PORTUGAL: Faro: 36°,58'N/07°,58'W

30. Adriatic Sea - ITALY: Alberoni: 45°,21’N/12°,19’E; Caorle 45°,35’N/12°,52’E; Punta Sabbioni: 45°,25’N/12°,41’E; San Nicolò: 45°,23’N/12°,21’E; Francavilla al Mare: 42°,21’N/14°,17’E; Foce dell´Isonzo: 45°,45’N/13°,31’E; Miramare: 45°,41’N/13°,43’E; Grotta del Sale: 42°,06’N/15°,29’E

31. Aegean Sea - GREECE: Edem: 37°,54'N/23°,42'; Tripiti: 40°,21'N/23°,53'E; Amnissos W: 35°,19'N/25°,11'E; Matala: 34°,59'N/24°,45'E; Lindos: 36°,04'N/28°,06'E

34. Ionian Sea - ITALY: Porto Colombo: 41°,05’N/17°,05’E; Cala Incina: 40°,57’N/17°,18’E; Cala Paura: 41°,00’N/17°,13’E; San Cataldo: 40°,22’N/18°,18’E; S. M. di Leuca: 39°,47’N/18°,18’E; San Isidoro: 40°,09’N/17°,57’E; Torre Vado: 39°,49’N/18°,15’E; Punta Braccetto: 36°,49’N/14°,28’E; Golfo di Dragunara: 40°,35’N/08°,11’E; Baia di Santa Reparata: 41°,15’N/09°,09’E

35. Western Mediterranean - ITALY: San Ambrogio: 38°,02’N/14°,04’E; San Vito lo Capo: 38°,10’N/12°,45’E; Isola Gallinara: 44°,02’N/08°,17’E; Genova: 44°,25’N/08°,56’E; Portovenere: 44°,02’N/09°,49’E; Bagno Gorgona MP: 43°,44’N/10°,16’E; Scalo Morosini: 43°,32’N/10°,17’E; Bagni Pancaldi: 43°,32’N/10°,17’E; Marina di Pisa: 43°,44’N/10°,16’E; Favignana: 37°,56’N/12°,20’E; Bagnara: 41°,03’N/13°,53’E; Via Caracciolo: 40°,50’N/14°,15’E; Palazzo Donn’Anna: 40°,49’N/14°,13’E; Lido di Fusaro: 40°,50’N/14°,01’E; Licola: 40°,54’N/14°,02’E; Mergellina: 40°,50’N/14°,14’E; Capo di Posillipo: 40°,47’N/14°,12’E; Golfo di Pozzuoli: 40°,48’N/14°,06’E; Villa Rosebery: 40°,47’N/14°,12’E; Punta del Capel Rosso: 42°,19’N/10°,55’E; Chiaia: 40°,43’N/13°,51’E; Citara: 40°,44’N/13°,54’E; P. Fetovaia: 42°,43’N/10°,08’E; Zuccale: 42°,45’N/10°,21; Cala Liberotto: 40°,26’N/09°,50’E; Spiaggia di S. Agostino: 41°,13’N/13°,30’E

40. Gulf of Maine/Bay of Fundy - UNITED STATES OF AMERICA: Duxbury I: 43°,00'N/70°,40'W; Plum Island S: 42°,41'N/70°,46'W.

41. Virginian - UNITED STATES OF AMERICA: Woodneck Beach: 41°,35'N/70°,39'W; Indian River Inlet Inside: 38°,36'N/75°,04'W; Indian River Inlet Outside: 38°,36'N/75°,04'W; Roosevelt Inlet Inside: 38°,47'N/75°,09'W40.

42. Carolinian - UNITED STATES OF AMERICA: DU Marine Lab: 34°,43'N/76°,40'W; Debidue: 33°,20'N/79°,10'W; St. Augustine Beach: 29°,53'N/81°,17'W;

43. Northern Gulf of Mexico - UNITED STATES OF AMERICA: Pine Beach: 30°,14'N/87°,44'W; Galveston Island: 29°,11'N/94°,57'W; Matakesset Beach Inside: 41°,21'N/70°,29'W; Panama City Beach: 30°,06'N/85°,46'W; Gulf - Cape San Blas SW: 29°,40'N/85°,23'W; St. George Island W: 29°,37'N/84°,52'W; Vero Beach: 27°,39'N/80°,21'W; - Anna Marie Inside: 27°,31'N/82°,44'W; Gasparilla Island PM: 26°,43'N/82°,15'W; Honeymoon Key O: 28°,03'N/82°,49'W; Manasota Beach: 27°,00'N/82°,24'W.

44. Black Sea - BULGARIA: Varna: 43°,12'N/27°,57'E; ROMANIA: Constanta: 44°,10'N/28°,39'E; Mamia: 44°,15'N/28°,37'E; Capul Midia: 44°,21'N/28°,41'E

63. Bahamian - UNITED STATES OF AMERICA: Little Duck Key: 24°,40'N/80°,13'W;

70. Floridian - UNITED STATES OF AMERICA: Bahia Honda Key SE: 24°,39'N/81°,15'W; Bahia Honda Key SW: 24°,39'N/81°,16'W; Duvall St. Fine: 24°,35'N/81°,50'W; Duvall St. Coarse: 24°,35'N/81°,50'W;

87. Northern and Central Red Sea - EGYPT: Cleopatra: 31°,12'N/29°,56'E; Markaz: 30°,53'N/28°,51'E; Sharm el-Arab I: 26°,03'N/33°,54'E; M Bareika W: 27°,45'N/34°,12'E; Giftun IsSS: 27°,10'N/33°,57'E; Nabq S: 28°,04'N/34°,25'E; Ras Nasrani: 27°,56'N/34°,23'E; Nuweiba: 28°,58'N/34°,39'E; Ras Sudr: 29°,36'N/32°,40'E; ISRAEL: Coral Beach S: 29°,29'N/34°,54'E

93. Central Somali Coast - SOMALIA: Elad: 02°,08'N/-45°,35'W

180. Southeastern Brazil - BRAZIL: Praia Martim de Sá: 23°,37'S/45°,23'W; Prumirim Beach: 23°,22'S/44°,57'W; Segredo Beach: 23°,49'S/45°,25'W

*Aspidiophorus paramediterraneus* Hummon, 1974

Terra typica: Piver’s Island, Carteret County, North Carolina, United State of America: 34°,43'N/76°,40'W

Record numbers: ENGLAND: (7); GERMANY: (1); SCOTLAND: (7); CHANNEL ISLES: (3); IRELAND: (1); ISLE OF MAN: (2); WALES: (1); FRANCE: (11); ITALY: (66); GREECE: (11); CYPRUS: (1); EGYPT: (12); ISRAEL: (1); UNITED STATES OF AMERICA: (34); THE CARIBBEAN ISLANDS: (3); MEXICO: (1); SOMALIA: (1); ECUADOR: (1) BRAZIL: (3); total: Wo: (164); Br: (3)

25. North Sea - ENGLAND: Bognor Regis: 50°,45'N/00°,42'W; GERMANY: Loreley Bank: 54°,12'N/08°,00'E; SCOTLAND: Fetlar Island: 60°,36'N/00°,56'W; Fetlar Island: 60°,36'N/00°,56'W;

26. Celtic Seas - CHANNEL ISLES: Corbiere Point: 49°,10'N/02°,16'W; Gorey: 49°,11'N/02°,02'W; Petit Point: 49°,25'N/02°,32'W: SCOTLAND: Firemore Beach A: 57°,49'N/05°,41'W; Firemore Beach D: 57°,49'N/05°,41'W; Irvine Bay A: 55°,37'N/04°,44'W; Kirkcolm: 54°,58'N/05°,04'W; Monifieth: 56°,28'N/02°,49'W; IRELAND: Ballyrisode Strand: 51°,30'N/09°,39'W; ENGLAND: Braunton Marsh: 51°,04'N/04°,10'W; Cove: 49°,53'N/06°,20'W; Old Quay Flats: 49°,57'N/06°,17'W; Pentle Bay: 49°,56'N/06°,19'W; Porthmellon: 49°,54'N/06°,18'W; Studland: 50°,35'N/01°,56'W; FRANCE: Ile de Batz: 48°,44'N/04°,00'W; Banc de Duslen/ Pighet: 48°,43'N/03°,58'W; Aber de Roscoff: 48°,42'N/04°,00'W; Lab de Roscoff: 48°,43'N/03°,59'W; ISLE OF MAN: Derby Haven: 54°,05'N/04°,39'W; Port Erin S: 54°,05'N/04°,45'W; WALES: Llanbedrog: 52°,51'N/04°,34'W

27. South European Atlantic Shelf - FRANCE: Plage d'Eyrac: 44°,39'N/01°,10'W

30. Adriatic Sea - ITALY Cala delle Arene: 42°,07’N/15°,30’E; Grotta del Sale: 42°,06’N/15°,29’E; Grotta delle Viole: 42°,06’N/15°,29’E; Puglia - Grotto di Cala Corvino: 40°,58’N/17°,18’E; Cozze: 41°,02’N/17°,08’E; Torre Guaceto: 40°,43’N/17°,48’E; Lido Porto Nuovo: 41°,51’N/16°,11’E; San Cataldo: 40°,22’N/18°,18’E; Costa N-E: 42°,07’N/15°,31’E;Francavilla al Mare: 42°,21’N/14°,17’E; Montesilvano Marina: 42°,25’N/14°,09’E; Pesaro: 43°,58’N/12°,55’E; Cala di Turchi: 42°,09’N/15°,30’E

31. Aegean Sea - GREECE: Akroteri Red: 36°,17'N/25°,22'E; Kehres: 37°,55'N/23°,01'E; Krassa (=Banana): 39°,08'N/23°,23'E; Loutsa: 37°,56'N/24°,01'E; Varkiza: 37°,48'N/23°,48'E; Ierissos: 40°,22'N/23°,52'E; Glystra: 36°,02'N/28°,00'E; Lindos: 36°,04'N/28°,06'E; Agios Minas: 36°,21'N/27°,57'E; Tsambika: 36°,12'N/28°,09'E; Plakias: 35°,11'N/24°,23'E.

32. Levantine Sea - CYPRUS: Protaras: 34°,58'N/34°,04'E; EGYPT: Hannoville (‘Agami): 31°,06'N/29°,43'E; Betash (‘Agami) 31°,06'N/29°,43'E; Abu Qir, 31°,18'N/30°,02'E; el-Arish E: 31°,08'N/33°,43'E; el-Arish W: 31°,07'N/33°,41'E; el-Arish EO: 31°,07'N/33°,42'E; Cleopatra, 31°,12'N/29°,56'E; Mersa Matruh; 31°,20'N/27°,14'E

34. Ionian Sea - ITALY: Marina di Ginosa: 40°,25’N/16°,49’E; Giardini Noxos: 37°,50’N/15°,17’E; San Isidoro: 40°,09’N/17°,57’E; Torre Vado: 39°,49’N/18°,15’E; Baia Verde: 40°,00’N/18°,01’E

35. Western Mediterranean - FRANCE: Plage de Calvi: 42°,33'N/08°,46'E; Anse de Fautea: 41°,42'N/09°,24'E; Marine de Farinole: 42°,44'N/09°,20'E; Marine de Pietracorbara: 42°,50'N/09°,24'E; Sagone: 42°,06'N/08°,41'E; Ile de Jarre: 43°,11'N/05°,21'E; ITALY: Albisola Marina: 44°,18’N/08°,29’E; Framura: 44°,07’N/09°,50’E; San Remo: 43°,48’N/07°,46’E; Torre al Bagno: 43°,02’N/09°,50’E; La Biodola: 42°,48’N/10°,15’E; Cavo: 42°,51’N/10°,25’E; Pomonte: 42°,47’N/10°,06’E; San Andrea: 42°,48’N/10°,07’E; Marina Donoratico: 43°,09’N/10°,40’E; Bagno Gorgona MP: 43°,44’N/10°,16’E; Bagni Pancaldi: 43°,32’N/10°,17’E; Baia di Anzachena: 41°,08’N/09°,28’E; Calasetta: 39°,07’N/08°,21’E; Portu la Rena: 40°,55’N/08°,43’E; Capo Turri: 38°,58’N/08°,26’E;ala Pisana: 35°,30’N/12°,37’E; Lacco Ameno: 40°,45’N/13°,53’E; Bagnetielli: 40°,44’N/13°,55’E; Casamicciola: 40°,44’N/13°,54’E; Chiaia: 40°,43’N/13°,51’E; Spiaggia degli Inglesi: 40°,45’N/13°,56’E; Spiaggia di Ischia Porto: 40°,45’N/13°,56’E; Anzio: 41°,31’N/12°,32’E; Hotel Ninfeo SSA: 41°,13’N/13°,30’E; Spiaggia di Serapo: 41°,12’N/13°,33’E; Terracina: 41°,17’N/13°,15’E; Arenella: 42°,22’N/10°,53’E; Cannelle: 42°,21’N/10°,54’E; Via Caracciolo: 40°,50’N/14°,15’E; Palazzo Donn’Anna: 40°,49’N/14°,13’E; Lido di Fusaro: 40°,50’N/14°,01’E; Licola: 40°,54’N/14°,02’E; Castel dell’Ovo: 40°,50’N/14°,16’E; Palinuro: 40°,00’N/15°,15’E; Capo di Posillipo: 40°,47’N/14°,12’E; Golfo di Pozzuoli: 40°,48’N/14°,06’E; Torregaveta: 40°,47’N/14°,02’E; Tombolo di Feniglia: 42°,24’N/11°,14’E; Mortelliccio: 42°,56’N/10°,41’E; Punta Ala: 42°,49’N/10°,44’E; P. Fetovaia: 42°,43’N/10°,08’E; Zuccale: 42°,45’N/10°,21’E; Marina di Gairo: 39°,45’N/09°,41’E; Porto Sardinia: 41°,09’N/09°,28’E; Chiaia di Luna: 40°,54’N/12°,57’E; Cala Nave: 40°,47’N/13°,36’E; Lido di Procida: 40°,44’N/14°,00’E; S.M. Navarrese: 39°,58’N/09°,41’E; San Vito lo Capo: 38°,10’N/12°,45’E

40. Gulf of Maine/Bay of Fundy - UNITED STATES OF AMERICA: Saquish Neck I: 43°,00'N/70°,40'W; Singing Beach: 42°,34'N/70°,45'W; Griffiths Head RSP: 43°,46'N/69°,43'W

41. Virginian - UNITED STATES OF AMERICA: Cape Henlopen Bight: 38°,47'N/75°,05'W; Crane’s Beach: 41°,32'N/70°,41'W; Egypt Beach: 41°,45'N/70°,26'W

42. Carolinian - UNITED STATES OF AMERICA: Shackleford East: 34°,41'N/76°,38'W; Shackleford Point West: 34°,41'N/76°,39'W; DU Marine Lab: 34°,43'N/76°,40'W; Myrtle Beach: 33°,39'N/78°,55'W

43. Northern Gulf of Mexico - UNITED STATES OF AMERICA: Pine Beach: 30°,14'N/87°,44'W; Destin Beach: 30°,23'N/86°,29'W; Grayton Beach: 30°,19'N/86°,09'W; Pensacola Beach: 30°,17'N/87°,08'W; St. George Island E: 29°,41'N/84°,44'W; St. George Island W: 29°,37'N/84°,52'W.

63. Bahamian - UNITED STATES OF AMERICA: Little Duck Key: 24°,40'N/80°,13'W

64. Eastern Caribbean - THE CARIBBEAN ISLANDS:Magens Bay: 18°,22'N/64°,55'W; Lindbergh Bay: 18°,20'N/64°,58'W; Muller Bay: 18°,20'N/64°,51'W

68. Western Caribbean - MEXICO: Cancún Island: 21°,05'N/86°,47'W

70. Floridian - UNITED STATES OF AMERICA: Anna Marie Inside: 27°,31'N/82°,44'W ; Delnor-Wiggins SRA: 26°,17'N/81°,49'W; Egmont Key NE: 27°,35'N/82°,45'W; Egmont Key NW: 29°,13'N/85°,24'W; Egmont Key SE: 27°,35'N/82°,45'W; Englewood Beach: 26°,57'N/82°,23'W; Gasparilla Island PM: 26°,43'N/82°,15'W; Gasparilla Island RA 26°,44'N/82°,15'W; Honeymoon Key O: 28°,03'N/82°,49'W; Mullet Key NO: 27°,38'N/82°,44'W; Venice Beach: 27°,05'N/82°,23'W; Adams Key: 25°,24'N/80°,14'W; Elliott Key Dock: 25°,26'N/80°,12'W; Bahia Honda Key SE: 24°,39'N/81°,15'W; Bahia Honda Key NW: 24°,39'N/81°,16'W; Big Pine Key STB: 24°,39'N/81°,20'W; Sombrero Beach: 24°,41'N/81°,05'W.

87. Northern and Central Red Sea - EGYPT: Daghashland: 29°,26'N/32°,45'E; Nuweiba: 28°,58'N/34°,39'E; Sharm el-Sheikh: 27°,50'N/34°,15'E; Uyun Musa: 29°,52'N/32°,38'E; ISRAEL: Kiryat Yam: 31°,52'N/35°,03'E.

93. Central Somali Coast - SOMALIA: Gesira: 01°,57'N/45°,11'W

152. Hawaii - UNITED STATES OF AMERICA: Kaiona Beach: 21°,19'N/157°,41'W.

171. Guayaquil - ECUADOR: Jambeli Island O: 02°,14'S/80°,14'W

180. Southeastern Brazil - BRAZIL: Castelhanos Beach: 23°,51'S/45°,17'W; Grande Beach: 23°,51'S/45°,25'W; Segredo Beach: 23°,49'S/45°,25'W

*Aspidiophorus tentaculatus* Wilke, 1954

Terra typica: Capo di Posillipo, Napoli, Campania, Italy: 40°,47'N/14°,12'E

Record numbers: GREECE: (4); ITALY: (7); CAYMAN ISLAND: (1); EGYPT: (7); ISRAEL: (6); UNITED STATES OF AMERICA: (6); SOMALIA: (1); BRAZIL (3); total: Wo: (32); Br: (3)

31. Aegean Sea - GREECE: Monolithos: 36°,20'N/25°,29'E; Perissa: 36°,16'N/25°,27'E; Ierissos: 40°,22'N/23°,52'E; Vai: 35°,14'N/26°,16'E

35. Western Mediterranean - ITALY: Via Caracciolo: 40°,50’N/14°,15’E; Castel dell’Ovo: 40°,50’N/14°,16’E; Capo di Posillipo: 40°,47’N/14°,12’E; Villa Rosebery: 40°,47’N/14°,12’E; Casamicciola: 40°,44’N/13°,54’E; Chiaia: 40°,43’N/13°,51’E; Cala Scirocco: 42°,20’N/10°,20’E

65. Greater Antilles - CAYMAN ISLAND: Grape Tree Bay 19°,41’N/ 80°03’W

87. Northern and Central Red Sea - EGYPT: Giftun IsSE: 27°,10'N/33°,57'E; Main Beach RM: 27°,44'N/34°,12'E; Middle Garden: 27°,54'N/34°,21'E; Moon Beach: 29°,14'N/32°,53'E; Moon Valley: 27°,15'N/33°,48'E; Ras Sudr: 29°,36'N/32°,40'E; Tareef el-Reeh S: 28°,49'N/34°,36'E; ISRAEL: Coral Beach M2: 29°,30'N/34°,55'E; Coral Beach M3: 29°,30'N/34°,55'E; Coral Beach M4: 29°,30'N/34°,55'E; Coral Beach M5: 29°,30'N/34°,55'E; Coral Beach N1: 29°,30'N/34°,55'E; Princess Hotel: 29°,29'N/34°,54'E

70. Floridian - UNITED STATES OF AMERICA: Monroe - Bahia Honda Key SE: 24°,39'N/81°,15'W; Bahia Honda Key NW: 24°,39'N/81°,16'W; Crandon Park N: 25°,43'N/80°,09'W; Crandon Park Outside: 25°,43'N/80°,09'W; Elliott Key Dock: 25°,26'N/80°,12'W; Matheson Hammock Beach: 25°,40'N/80°,15'W

93. Central Somali Coast - SOMALIA: Gesira: 01°,57'N/45°,11'W

180. Southeastern Brazil - BRAZIL: Prumirim Island: 23°,21'S/44°,56'W; Vermelha Beach: 23°,25'S/45°,02'W; Fome Beach: 23°44'27.9"S 45°16'00.9"W

**Genus** *Chaetonotus* Ehrenberg, 1830

*Chaetonotus (Chaetonotus) apechochaetus* Hummon, Balsamo and Todaro, 1992

Terra typica: Marina di Ginosa, Taránto, Puglia, Italy: 40°,25'N/16°,49'E

Record numbers: ITALY: (4); GREECE: (10); CYPRUS: (2); EGYPT: (8); ISRAEL: (4); FRANCE: (27); BRAZIL: (3); total: Wo: (55); Br: (3)

30. Adriatic Sea - ITALY: Costa N-O: 42°,08’N/15°,31’E; 42°,08’N/15°,31’E

31. Aegean Sea - GREECE: Akroteri White: 36°,17'N/25°,21'E; Monolithos: 36°,20'N/25°,29'E; Kehres: 37°,55'N/23°,01'E; Loutsa: 37°,56'N/24°,01'E; Varkiza; 37°,48'N/23°,48'E; Sani: 40°,05'N/23°,18'E Ialiosos: 36°,23'N/28°,10'E; Lindos: 36°,04'N/28°,06'E; Tsambika: 36°,12'N/28°,09'E; Matala: 34°,59'N/24°,45'E

32. Levantine Sea - CYPRUS: Agia Napa: 34°,58'N/33°,59'E; Yeroskipos (=Geroskipos): 34°,43'N/32°,25'E; EGYPT: el-Arish EO: 31°,07'N/33°,42'E; Cleopatra: 31°,12'N/29°,56'E; ISRAEL: Coral Beach M: 29°,30'N/34°,55'E; Coral Beach N1: 29°,30'N/34°,55'E; North Beach: 29°,32'N/34°,57'E; Princess Hotel: 29°,29'N/34°,54'E

34. Ionian Sea - ITALY: Marina di Ginosa 40°,25’N/16°,49’E; Torre San Giovanni: 39°,53’N/18°,07’E

35. Western Mediterranean - FRANCE: Plage de Calvi: 42°,33'N/08°,46'E; Anse de Fautea: 41°,42'N/09°,24'E; Marine de Farinole: 42°,44'N/09°,20'E; Plage du Padulone: 42°,06'N/09°,33'E; Sagone: 42°,06'N/08°,41'E; ITALY: Torre al Bagno: 43°,02’N/09°,50’E; Cala della Mortola: 43°,04’N/09°,50’E; Seno della Perruccia: 43°,02’N/09°,47’E; Cavo: 42°,51’N/10°,25’E; San Andrea: 42°,48’N/10°,07’E; Genova: 44°,25’N/08°,56’E; San Remo: 43°,48’N/07°,46’E; Isola Gallinara: 44°,02’N/08°,17’E; Bagno Gorgona MP: 43°,44’N/10°,16’E; Marina di Pisa: 43°,44’N/10°,16’E; Baia di Anzachena: 41°,08’N/09°,28’E; Masua: 39°,19’N/09°,35’E; Baia di Santa Reparata: 41°,15’N/09°,09’E; Punta Braccetto: 36°,49’N/14°,28’E; Porto Palo: 37°,35’N/12°,53’E; Punta Ala: 42°,49’N/10°,44’E; Cannelle: 42°,21’N/10°,54’E; Punta del Capel Rosso: 42°,19’N/10°,55’E ; Casamicciola: 40°,44’N/13°,54’E; Spiaggia degli Inglesi: 40°,45’N/13°,56’E; Cala Nave: 40°,47’N/13°,36’E; Lido di Procida: 40°,44’N/14°,00’E

87. Northern and Central Red Sea - EGYPT: Shaab Abu Ramada 1: 27°,13'N/33°,52'E; Shaab Abu Ramada 2: 27°,13'N/33°,52'E; Shaab Abu Ramada 3: 27°,13'N/33°,52'E; M Bareika N: 27°,46'N/34°,12'E; Giftun IsSE: 27°,10'N/33°,57'E; Moon Valley: 27°,15'N/33°,48'E

180. Southeastern Brazil - BRAZIL: Prumirim Island: 23°,21'S/44°,56'W; Segredo Beach: 23°,49'S/45°,25'W; Rancho Beach: 23°20'55.0"S 44°43'15.7"W

*Chaetonotus (Schizochaetonotus) atrox* Wilke, 1954

Terra typica: Via Caracciolo, Napoli, Campania, Italy: 40°,50'N/14°,15'E

Record numbers: DENMARK: (1); GERMANY: (3); SWEDEN: (1); BRITISH ISLES: (1); WALES: (1); ENGLAND: (2); FRANCE: (6); PORTUGAL: (1); ITALY: (37); GREECE: (11); CYPRUS: (2); EGYPT: (21); ISRAEL: (3); UNITED STATES OF AMERICA: (13); INDIA: (8); JAPAN: (3); BRAZIL: (4); total: Wo: (114); Br: (4)

25. North Sea - DENMARK: Romo: 55°,04'N/08°,33'E; GERMANY: Felswatt: 54°,11'N/07°,51'E; Litoral station: 55°,01'N/08°,26'E; Uthörn: 55°,02'N/08°,26'E ; SWEDEN: Gulmarsfjord: 58°,20'N/11°,30'E

26. Celtic Seas - BRITISH ISLES: Corbiere Point: 49°,10'N/02°,16'W; WALES: Porth Trecastell: 53°,11'N/04°,34'W; ENGLAND: Bar Point: 49°,56'N/06°,18'W; Old Quay Flats: 49°,57'N/06°,17'W; FRANCE: Ile de Batz: 48°,44'N/04°,00'W; Banc de Duslen/Pighet: 48°,43'N/03°,58'W; Aber de Roscoff: 48°,42'N/04°,00'W

29. Azores Canaries Madeira - PORTUGAL: Porto Pim: 38°,30'N/28°,17'W

30. Adriatic Sea - ITALY: Punta Falconetto: 42°,08’N/15°,31’E; Foce dell’Isonzo: 45°,45’N/13°,31’E; Numana: 43°,25’N/13°,36’E; San Cataldo: 40°,22’N/18°,18’E; Grotto della Principessa: 39°,48’N/18°,22’E; Termoli: 42°,00’N/14°,59’E; Grotta delle Viole: 42°,06’N/15°,29’E

31. Aegean Sea - GREECE: Akroteri White: 36°,17'N/25°,21'E; Perissa: 36°,16'N/25°,27'E; Kehres: 37°,55'N/23°,01'E; Loutsa: 37°,56'N/24°,01'E; Varkiza: 37°,48'N/23°,48'E; Lindos: 36°,04'N/28°,06'E; Agios Minas: 36°,21'N/27°,57'E; Tsambika: 36°,12'N/28°,09'E; Makryalos: 35°,02'N/25°,57'E; Matala: 34°,59'N/24°,45'E; Vai: 35°,14'N/26°,16'E.

32. Levantine Sea - CYPRUS: Coral Bay: 34°,50'N/32°,21'E; Yeroskipos (=Geroskipos): 34°,43'N/32°,25'E; EGYPT: el-Arish E: 31°,08'N/33°,43'E; el-Arish W: 31°,07'N/33°,41'E; el-Arish EO: 31°,07'N/33°,42'E; Bir Mesud: 31°,14'N/29°,58'E; Boghoush W: 31°,16'N/27°,49'E; Cleopatra: 31°,12'N/29°,56'E; Mamura: 31°,17'N/30°,01'E; Mersa Matruh: 31°,20'N/27°,14'E; Riese: 31°,08'N/33°,44'E; ISRAEL: Coral Beach M3: 29°,30'N/34°,55'E; Coral Beach M4: 29°,30'N/34°,55'E; Coral Beach N1: 29°,30'N/34°,55'E

34. Ionian Sea - ITALY: Brancaleone: 37°,58’N/16°,07’E; Torre Vado: 39°,49’N/18°,15’E; Baia Verde: 40°,00’N/17°,59’E

35. Western Mediterranean - FRANCE: Plage de Calvi: 42°,33'N/08°,46'E; Moriani Plage: 42°,22'N/09°,31'E; Ile Riou: 43°,09'N/05°,22'E; ITALY: Albisola Marina: 44°,18’N/08°,29’E; Genova: 44°,25’N/08°,56’E; San Remo: 43°,48’N/07°,46’E; Bagno Gorgona MP: 43°,44’N/10°,16’E; Secche delle Meloria: 43°,32’N/10°,16’E; Marina di Pisa: 43°,44’N/10°,16’E: Seno della Perruccia: 43°,02’N/09°,47’E; Sardinia - Calasetta: 39°,07’N/08°,21’E; Capo Turri: 38°,58’N/08°,26’E; Bagnetielli: 40°,44’N/13°,55’E; Casamicciola: 40°,44’N/13°,54’E; Chiaia: 40°,43’N/13°,51’E; Citara: 40°,44’N/13°,54’E; Spiaggia degli Inglesi: 40°,45’N/13°,56’E; Via Caracciolo: 40°,50’N/14°,15’E; Cuma: 40°,52’N/14°,01’E; Palazzo Donn’Anna: 40°,49’N/14°,13’E; Paestum: 40°,25’N/14°,58’E; Palinuro: 40°,00’N/15°,15’E; Capo di Posillipo: 40°,47’N/14°,12’E; Torregaveta: 40°,47’N/14°,02’E; Punta del Fenaio: 42°,24’N/10°,53’E; Tombolo di Feniglia: 42°,24’N/11°,14’E; Mortelliccio: 42°,56’N/10°,41’E; Cala Nave: 40°,47’N/13°,36’E; Porto Sardinia: 41°,09’N/09°,28’E; Spiaggia di Serapo: 41°,12’N/13°,33’E

41. Virginian - UNITED STATES OF AMERICA: Ship Bottom: 39°,38'N/74°,11'W

43. Northern Gulf of Mexico - UNITED STATES OF AMERICA: FSU Marine Lab: 29°,50'N/84°,34'W; Honeymoon Key O: 28°,03'N/82°,49'W; Panama City Beach: 30°,06'N/85°,46'W; Cape San Blas SW: 29°,40'N/85°,23'W; St. George Island E: 29°,41'N/84°,44'W; St. George Island W: 29°,37'N/84°,52'W.

70. Floridian - UNITED STATES OF AMERICA: - Miami Beach: 25°,47'N/80°,07'W; Wiggins SRA: 26°,17'N/81°,49'W; Crandon Park Outside: 25°,43'N/80°,09'W; Elliott Key Dock: 25°,26'N/80°,12'W; Sands Key: 25°,30'N/80°,11'W; Duvall St. Coarse: 24°,35'N/81°,50'W

87. Northern and Central Red Sea - EGYPT: Shaab Abu Ramada 2: 27°,13'N/33°,52'E; Sharm el-Arab O: 26°,03'N/33°,54'E; Wadi `Araba: 29°,08'N/32°,39'E; M. Bareika W: 27°,45'N/34°,12'E; Giftun IsSE: 27°,10'N/33°,57'E; Giftun VSI: 27°,10'N/33°,49'E; Giftun VSO: 27°,10'N/33°,49'E; Moon Valley: 27°,15'N/33°,48'E; Na'ama Bay S: 27°,53'N/34°,19'E; Port Bay, Sharm: 27°,50'N/34°,14'E; Sharm el-Sheikh: 27°,50'N/34°,15'E; Tareef el-Reeh: 28°,50'N/34°,36'E; ISRAEL: Coral Beach M3: 29°,30'N/34°,55'E; Coral Beach M4: 29°,30'N/34°,55'E; Coral Beach N1: 29°,30'N/34°,55'E

105. Maldives – INDIA: Kavaratti Island: 10°,33'N/72°,38'E; Minicoy Island: 8°,17'N/73°,04'E

107. Eastern India - INDIA: Bahuda Estuary: 19°,30'N/84°,45'E; Gopalpur Beach: 19°,15'N/84°,55'E; Bhilimilipatnam: 17°,44'N/83°,23'E; Light House Point #5: 17°,41'N/83°,17'E

109. Andaman and Nicobar Islands – INDIA: Car Nicobar - Unassigned: 13°N/93°E; Hut Bay: 10°,38'N/92°,33'E

121. South Kuroshio - JAPAN: Iriomote Jima Sta. 2: 24°,10'N/123°,53'E; Ishigaki: 24°,09'N/124°,03'E; Okinawa Sta. 9: 26°,12'N/127°,39'E

180. Southeastern Brazil - BRAZIL: Castelhanos Beach: 23°,51'S/45°,17'W; Prumirim Island: 23°,22'S/44°,57'W; Vermelha Beach: 23°,25'S/45°,02'W; Segredo Beach; 23°,37'S/45°,23'W

*Chaetonotus (Schizochaetonotus) dispar* Wilke, 1954

Terra typica: Mergellina, Napoli, Campania, Italy: 40°,50'N/14°,14'E

Record numbers: FRANCE: (1); SCOTLAND: (1); FRANCE: (2); ITALY: (57); UNITED STATES OF AMERICA: (15); THE CARIBBEAN ISLANDS: (1) BRAZIL: (3); total: Wo: (77); Br: (3)

26. Celtic Seas - FRANCE: Ile Callot W: 48°,40'N/03°,56'W; SCOTLAND: Laggan: 56°,51'N/05°,49'W

30. Adriatic Sea - FRANCE: Marine de Pietracorbara: 42°,50'N/09°,24'E; Ile de Jarre: 43°,11'N/05°,21'E; ITALY: Alimini: 40°,12’N/18°,26’E; San Cataldo: 40°,22’N/18°,18’E; Punta Falconetto: 42°,08’N/15°,31’E; Fosse dell´Isonzo: 45°,45’N/13°,31’E; Miramare: 45°,41’N/13°,43’E; Cala Spino: 42°,07’N/15°,30’E

34. Ionian Sea - ITALY: Marina di Ginosa: 40°,25’N/16°,49’E; San Isidoro: 40°,09’N/17°,57’E; 40°,13’N/17°,54’E; Torre Lapillo: 40°,16’N/17°,51’E; Torre Vado: 39°,49’N/18°,15’E; Giardini Noxos: 37°,50’N/15°,17’E; Rada di Portopalo: 36°,40’N/15°,07’E; Grotta delle Viole: 42°,06’N/15°,29’E

35. Western Mediterranean - ITALY: Torre al Bagno: 43°,02’N/09°,50’E; Punta di Civitata: 43°,01’N/09°,49’E; Cala della Mortola: 43°,04’N/09°,50’E; Seno della Perruccia: 43°,02’N/09°,47’E; Punta del Recisello: 43°,03’N/09°,48’E; Cala dello Zurletto: 43°,03’N/09°,51’E; La Biodola: 42°,48’N/10°,15’E; Cavo: 42°,51’N/10°,25’E; San Andrea: 42°,48’N/10°,07’E; Bagno Gorgona MP: 43°,44’N/10°,16’E; Secche delle Meloria: 43°,32’N/10°,16’E; Bagni Pancaldi: 43°,32’N/10°,17’E; Spiaggia dei Conigli: 35°,30’N/12°,37’E; Cala Pisana: 35°,30’N/12°,37’E; Torre Corsari: 39°,41’N/08°,27’E; Golfo di Dragunara: 40°,35’N/08°,11’E; Lacco Ameno: 40°,45’N/13°,53’E; Casamicciola: 40°,44’N/13°,54’E; Chiaia: 40°,43’N/13°,51’E; Citara: 40°,44’N/13°,54’E; Grotta del Mago: 40°,32’N/14°,04’E; Ansedonia: 42°,24’N/11°,17’E; Bagnara: 41°,03’N/13°,53’E; Via Caracciolo: 40°,50’N/14°,15’E; Mergellina: 40°,50’N/14°,14’E; Castel dell’Ovo: 40°,50’N/14°,16’E; Paestum: 40°,25’N/14°,58’E; Palinuro: 40°,00’N/15°,15’E; Capo di Posillipo: 40°,47’N/14°,12’E; Golfo di Pozzuoli: 40°,48’N/14°,06’E; Villa Rosebery: 40°,47’N/14°,12’E; Torregaveta: 40°,47’N/14°,02’E; Marina di Campo: 42°,44’N/10°,17’E; P. Fetovaia: 42°,43’N/10°,08’E; Zuccale: 42°,45’N/10°,21’E; Cannelle: 42°,21’N/10°,54’E; Cala Gemelle: 42°,19’N/10°,18’E; Cala Maestra: 42°,21’N/10°,17’E; Cala Mendolina: 42°,19’N/10°,17’E; Lido di Procida: 40°,44’N/14°,00’E ; San Vito lo Capo: 38°,10’N/12°,45’E; San Fernando: 38°,30’N/15°,55’E; Cala Scirocco: 42°,20’N/10°,20’E; Spiaggia di Serapo: 41°,12’N/13°,33’E

41. Virginian - UNITED STATES OF AMERICA: Griffiths Head RSP: 43°,46'N/69°,43'W

43. Northern Gulf of Mexico - UNITED STATES OF AMERICA: Pine Beach: 30°,14'N/87°,44'W; Cape Henlopen Bight: 38°,47'N/75°,05'W; Okaloosa - Destin Beach: 30°,23'N/86°,29'W; Grayton Beach: 30°,19'N/86°,09'W; Navarre Beach: 30°,16'N/86°,55'W; Panama City Beach: 30°,06'N/85°,46'W; Pensacola Beach: 30°,17'N/87°,08'W

64. Eastern Caribbean - THE CARIBBEAN ISLANDS: Little Lameshur Bay: 18°,19'N/64°,44'W

70. Floridian - UNITED STATES OF AMERICA: Anna Marie Inside: 27°,31'N/82°,44'W; Adams Key: 25°,24'N/80°,14'W; Crandon Park N: 25°,43'N/80°,09'W; Crandon Park Inside: 25°,43'N/80°,09'W; Crandon Park Outside: 25°,43'N/80°,09'W; Elliott Key Dock: 25°,26'N/80°,12'W; Bahia Honda Key NW: 24°,39'N/81°,16'W

180. Southeastern Brazil - BRAZIL: Belga: 23°,52'S/45°,26'W; P. Grande: 23°,51'S/45°,25'W; Vermelha Beach: 23°,11'S/44°,38'W

*Chaetonotus (Schizochaetonotus) neptuni* Wilke, 1954

Terra typica: Capo di Posillipo, Napoli, Campania, Italy; 40°,47'N/14°,12'E

Record numbers: BELGIUM: (1); FRANCE: (3); GREECE: (4); CYPRUS: (1); EGYPT: (11); ITALY: (30); UNITED STATES OF AMERICA:(4); BRAZIL: (3); total: Wo: (54); Br: (3)

25. North Sea - BELGIUM: North Sea 430: 51°,32'N/02°,40'E

26. Celtic Seas - FRANCE: Banc de Duslen/Pighet: 48°,43'N/03°,58'W; Basse Plate: 48°,44'N/04°,02'W

30. Adriatic Sea - ITALY: Grotto di Cala Corvino: 40°,58’N/17°,18’E; San Cataldo: 40°,22’N/18°,18’E; Torre Guaceto: 40°,43’N/17°,48’E; Cala Spino: 42°,07’N/15°,30’E

31. Aegean Sea - GREECE: Glystra: 36°,02'N/28°,00'E; Ialiosos: 36°,23'N/28°,10'E; Lindos: 36°,04'N/28°,06'E; Matala: 34°,59'N/24°,45'E

32. Levantine Sea - CYPRUS: Coral Bay: 34°,50'N/32°,21'E; EGYPT: Abu Qir: 31°,18'N/30°,02'E; Cleopatra: 31°,12'N/29°,56'E; Mersa Matruh: 31°,20'N/27°,14'E

34. Ionian Sea - ITALY: S.M. di Leuca: 39°,47’N/18°,18’E; San Isidoro: 40°,09’N/17°,57’E; Torre Vado: 39°,49’N/18°,15’E

35. Western Mediterranean - ITALY: Torre al Bagno: 43°,02’N/09°,50’E; Punta del Recisello: 43°,03’N/09°,48’E; Porto Vecchio: 43°,04’N/09°,50’E; Genova: 44°,25’N/08°,56’E; Pomonte: 42°,47’N/10°,06’E; Bosa Marina: 40°,17’N/08°,29’E; Calasetta: 39°,07’N/08°,21’E; Golfo di Dragunara: 40°,35’N/08°,11’E; Portu la Rena: 40°,55’N/08°,43’E; San Teodoro: 40°,47’N/09°,41’E; Cala Pisana: 35°,30’N/12°,37’E; Punta Ala 42°,49’N/10°,44’E; Lacco Ameno: 40°,45’N/13°,53’E; Grotta delle Formiche: 40°,54’N/14°,10’E; Spiaggia degli Inglesi: 40°,45’N/13°,56’E; Via Caracciolo: 40°,50’N/14°,15’E; Donn’Anna: 40°,49’N/14°,13’E; Palinuro: 40°,00’N/15°,15’E; Capo di Posillipo: 40°,47’N/14°,12’E; Punta del Fenaio: 42°,24’N/10°,53’E; Cala Liberotto: 40°,26’N/09°,50’E; Cala Nave: 40°,47’N/13°,36’E; Zuccale: 42°,45’N/10°,21’E. FRANCE: Sagone: 42°,06'N/08°,41'E

42. Carolinian - UNITED STATES OF AMERICA: Cape Hatteras Sta.5: 34°,10'N/76°,39'W; Cape Hatteras Sta.9: 34°,34'N/76°,40W

70. Floridian - UNITED STATES OF AMERICA: Egmont Key SE: 27°,35'N/82°,45'W; Gasparilla Island PM: 26°,43'N/82°,15'W

87. Northern and Central Red Sea - EGYPT: Daghashland: 29°,26'N/32°,45'E; Giftun IsSE: 27°,10'N/33°,57'E; Giftun VSO: 27°,10'N/33°,49'E; Middle Garden: 27°,54'N/34°,21'E; Moon Valley: 27°,15'N/33°,48'E; Na'ama Bay N: 27°,53'N/34°,19'E; Nabq S: 28°,04'N/34°,25'E; Ras Nasrani: 27°,56'N/34°,23'E

180. Southeastern Brazil - BRAZIL: Castelhanos Beach: 23°,51'S/45°,17'W; Fome Beach: 23°44'27.9"S/45°16'00.9"W; Prumirim Island: 23°,21'S/44°,56'W

**Genus** *Halichaetonotus* Remane, 1936

*Halichaetonotus decipiens* (Remane, 1926)

Terra typica: Kiel, Germany: 54°N/10°E

Record numbers: FRANCE: (11); GERMANY: (2); ITALY: (2); GREECE: (1); EGYPT: (7); UNITED STATES OF AMERICA: (3); BULGARIA: (1); ROMANIA: (2); BRAZIL: (5); total: Wo: (29); Br: (5)

25. North Sea - FRANCE: Ambleteuse: 50°,48'N/01°,35'E; Hardelot: 50°,38'N/01°,34'E; Le Hourdel: 50°,12'N/01°,31'E; Onival: 50°,04'N/01°,23'E; Sangatte: 50°,56'N/01°,46'E; Wissant: 50°,53'N/01°,38'E; Courseules: 49°,18'N/00°,28'W; Luc-sur-Mer: 49°,16'N/00°,21'W; GERMANY: Felswatt of Hlgloland: 54°,11'N/07°,51'E; Kieler Bucht: 54°N/10°E

26. Celtic Seas - FRANCE: Ile Callot W: 48°,40'N/03°,56'W

27. South European Atlantic Shelf - FRANCE: Montalivet: 45°,26'N/01°,10'W

30. Adriatic Sea - ITALY: Torre Fortore: 41°,54’N/15°,21’E; Marche - Numana: 43°,25’N/13°,36’E

31. Aegean Sea - GREECE: Sitia: 35°,11'N/26°,06'E

32. Levantine Sea - EGYPT: Cleopatra: 31°,12'N/29°,56'E; Mamura: 31°,17'N/30°,01'E; Mersa Matruh: 31°,20'N/27°,14'E; Fâyid: 30°,19'N/32°,18'E

35. Western Mediterranean - FRANCE: Marine de Pietracorbara: 42°,50'N/09°,24'E; ITALY: Punta del Recisello: 43°,03’N/09°,48’E; Cala Pisana: 35°,30’N/12°,37’E; Fiumicino: 41°,47’N/12°,13’E; Pozzuoli: 40°,50’N/14°,06’E

40. Gulf of Maine/Bay of Fundy - UNITED STATES OF AMERICA: Plum Island SE: 42°,42'N/70°,46'W; Plum Island SW: 42°,42'N/70°,47'W

43. Northern Gulf of Mexico - UNITED STATES OF AMERICA: Padre Island S: 26°,06'N/97°,09'W

44. Black Sea: BULGARIA: Varna: 43°,12'N/27°,57'E; ROMANIA: Constanta: 44°,10'N/28°,39'E; Mamia: 44°,15'N/28°,37'E

87. Northern and Central Red Sea - EGYPT: Bareika N: 27°,46'N/34°,12'E; M Bareika W: 27°,45'N/34°,12'E; Nabq: 28°,05'N/34°,25'E

180. Southeastern Brazil - BRAZIL: Castelhanos Beach: 23°,51'S/45°,17'W; Grande Beach 23°,51'S/45°,25'W; Praia de Siriuba: 23°,45'S/45°,20'W; Praia Martim de Sá: 23°,37'S/45°,23'W; Segredo Beach: 23°,49'S/45°,25'W

*Halichaetonotus euromarinus* Hummon & Todaro 2010

Terra typica: Uthörn, Island Sylt, Germany: 55°,02'N/08°,26'E

Record numbers: GERMANY: (5); BELGIUM: (3); CHANNEL ISLES: (5); ENGLAND: (12); FRANCE: (3); IRELAND: (8); WALES: (4); SCOTLAND: (16); ISLE OF MAN: (1); FRANCE: (9); ITALY: (29); GREECE: (14); CYPRUS: (2); EGYPT: (17); ISRAEL: (10); UNITED STATES OF AMERICA: (29); BRAZIL: (2); total: Wo: (167); Br: (2)

25. North Sea - GERMANY: Lister Ley: 55°,01'N/08°,26'E; Munkmarsch: 54°,54'N/08°,22'E; Uthörn: 55°,02'N/08°,26'E; Lister Tief: 55°,04'N/08°,20'E; Loreley Bank: 54°,12'N/08°,00'E ; BELGIUM: Mariakerke: 51°,13'N/02°,53'E; Oostduinkerke: 51°,06'N/02°,34'E; Zwin: 51°,19'N/03°,21'E; CHANNEL ISLES: Gorey: 49°,11'N/02°,02'W; Greve du Lecq: 49°,14'N/02°,12'W; Ouaisne Bay: 49°,10'N/02°,11'W; Vazon Bay: 49°,28'N/02°,36'W; Petit Point: 49°,25'N/02°,32'W; ENGLAND: Brancaster: 52°,58'N/00°,29'E; Coresand Buoy: 50°,29'N/04°,09'W; Cove: 49°,53'N/06°,20'W; Hunstanton: 52°,56'N/00°,19'E; Paignton: 50°,23'N/03°,32'W; Seaton Carew: 54°,39'N/01°,11'W; Stoup Beck: 54°,26'N/00°,32'W; Thorp Bay: 51°,30'N/00°,40'E; Tynemouth: 55°,01'N/01°,26'W; Walton on the Naze: 51°,50'N/01°,07'E; Whitsand Bay W: 50°,19'N/04°,17'W; Whitsand Bay E: 50°,18'N/04°,14'W

26. Celtic Seas - FRANCE: Ambleteuse: 50°,48'N/01°,35'E; Courseules: 49°,18'N/00°,28'W; Banc de Duslen/Pighet: 48°,43'N/03°,58'W; IRELAND: Ballyrisode Strand: 51°,30'N/09°,39'W; Castle Gregory: 52°,15'N/10°,02'W; Inch Strand: 52°,07'N/09°,59'W; Keem Strand: 53°,57'N/10°,11'W; Trawmore Strand: 53°,57'N/10°,03'W; Waterville: 51°,50'N/10°,13'W; WALES: Borth: 52°,28'N/03°,56'W; Llanbedrog: 52°,28'N/03°,56'W; Morfa Nefyn: 52°,56'N/04°,39'W; Oxwich Beach: 51°,32'N/04°,04'W; SCOTLAND: Achnahaird: 58°,04'N/05°,18'W; Balnakeil: 58°,34'N/04°,43'W; Clashnessie: 58°,13'N/05°,20'W; Cruden Bay: 57°,24'N/01°,51'W; Farr Beach: 58°,31'N/04°,12'W; Firemore Beach A: 57°,49'N/05°,41'W; Firemore Beach D: 57°,49'N/05°,41'W; Gruinard Bay: 57°,51'N/05°,28'W; Irvine Bay A: 55°,37'N/04°,44'W; Lunan Bay: 56°,37'N/02°,30'W; Mellon Udrigle: 57°,53'N/05°,34'W; Oldshoremore: 58°,28'N/05°,08'W; Portlogan: 54°,43'N/04°,57'W; Rattray Head: 57°,34'N/01°,50'W; East Tongue: 58°,30'N/04°,22'W; Turnberry: 55°,18'N/04°,50'W; ISLE OF MAN: Port Erin N: 54°,05'N/04°,46'W; NORTHERN IRELAND: Glenariffe: 55°,03'N/06°,02'W; Whitepark Bay: 55°,14'N/06°,24'W

27. South European Atlantic Shelf - FRANCE: Plage d'Eyrac: 44°,39'N/01°,10'W; L´Herbe: 44°,42'N/01°,15'W; Petit Nice: 44°,33'N/01°,17'W; Le PiQuay: 44°,42'N/01°,15'W; Pyla-sur-Mer: 44°,38'N/01°,13'W

30. Adriatic Sea - ITALY: Grotto della Principessa: 39°,48’N/18°,22’E; Alberoni: 45°,21’N/12°,19’E; Eraclea Mare: 45°,32’N/12°,45’E; Foce dell’Isonzo: 45°,45’N/13°,31’E; Bibione: 45°,37’N/13°,02’E; Miramare: 45°,41’N/13°,43’E; Lido Porto Nuovo: 41°,51’N/16°,11’E; San Cataldo: 40°,22’N/18°,18’E; Siponto: 41°,37’N/15°,54’E; Vieste: 41°,52’N/16°,10’E

31. Aegean Sea - GREECE: Akroteri Red: 36°,17'N/25°,22'E; Monolithos: 36°,20'N/25°,29'E; Kehres: 37°,55'N/23°,01'E; Krassa (=Banana): 39°,08'N/23°,23'E; Loutsa: 37°,56'N/24°,01'E; Marathon: 38°,08'N/24°,02'E; Sani: 40°,05'N/23°,18'E; Tripiti: 40°,21'N/23°,53'E; Vourvourou: 40°,12'N/23°,47'E; Ierissos: 40°,22'N/23°,52'E; 35°,21'N/24°,19'E; Matala: 34°,59'N/24°,45'E; Pachia Ammos: 35°,06'N/25°,48'E; Plakias: 35°,11'N/24°,23'E; Tsambika: 36°,12'N/28°,09'E

32. Levantine Sea - CYPRUS: Agia Napa: 34°,58'N/33°,59'E; Coral Bay: 34°,50'N/32°,21'E; EGYPT: Aghiba: 31°,23'N/27°,00'E; Aida Hotel: 30°,50'N/29°,15'E; el-Arish E: 31°,08'N/33°,43'E; el-Arish W: 31°,07'N/33°,41'E; el-Arish EO: 31°,07'N/33°,42'E; Atic Hotel: 30°,48'N/29°,03'E; Betash (‘Agami): 31°,06'N/29°,43'E; Bir Mesud: 31°,14'N/29°,58'E; Boghoush E: 31°,16'N/27°,49'E; Green Beach: 31°,03'N/29°,41'E; Hannoville (‘Agami): 31°,06'N/29°,43'E; Mamura: 31°,17'N/30°,01'E; Marakia Resort: 30°,54'N/29°,29'E; Mersa Matruh: 31°,20'N/27°,14'E; Obaeid: 31°,24'N/27°,02'E; Abd el'Rahman: 30°,59'N/28°,42'E; Riese: 31°,08'N/33°,44'E; ISRAEL: Ahziv: 33°,02'N/35°,05'E; Ashkelon: 31°,40'N/34°,34'E; Ashdod: 31°,48'N/34°,38'E; Carmel TN: 32°,43'N/34°,56'E; Carmel TS: 32°,43'N/34°,56'E; Netanya N: 32°,19'N/34°,51'E; Nitzanim: 31°,44'N/34°,36'E; Palmachim N: 31°,53'N/34°,41'E; Palmachim S: 31°,53'N/34°,41'E; Tel Aviv: 32°,04'N/34°,45'E

35. Western Mediterranean - FRANCE: Marine de Farinole: 42°,44'N/09°,20'E ITALY: La Biodola: 42°,48’N/10°,15’E; Diano Marina: 43°,54’N/08°,05’E; Punta del Recisello: 43°,03’N/09°,48’E; Manfria: 37°,06’N/14°,06’E; Porto Palo: 37°,35’N/12°,53’E; Cala Sabina: 41°,02’N/09°,33’E; Baia di Santa Reparata: 41°,15’N/09°,09’E; Bagnara 41°,03’N/13°,53’E; Lido di Fusaro: 40°,50’N/14°,01’E; Paestum: 40°,25’N/14°,58’E; Golfo di Pozzuoli: 40°,48’N/14°,06’E; Torregaveta: 40°,47’N/14°,02’E; Casamicciola: 40°,44’N/13°,54’E; Chiaia: 40°,43’N/13°,51’E; Mortelliccio: 42°,56’N/10°,41’E; Porto Sardinia: 41°,09’N/09°,28’E; Ladispoli: 41°,57’N/12°,03’E; Torre Scissura: 41°,14’N/13°,29’E; Spiaggia di Serapo: 41°,12’N/13°,33’E

40. Gulf of Maine/Bay of Fundy - UNITED STATES OF AMERICA: Plum Island S: 42°,41'N/70°,46'W; Salisbury: 42°,50'N/70°,48'W; Situate: 42°,11'N/70°,42'W

41. Virginian - UNITED STATES OF AMERICA: Nobska Beach M: 41°,31'N/70°,40'W; Nobska Beach W: 41°,31'N/70°,40'W; Virginia Beach: 36°,52'N/75°,58'W

42. Carolinian - UNITED STATES OF AMERICA: Beaufort Inlet Green Out: 34°,40'N/76°,40'W; Shackleford East: 34°,41'N/76°,38'W

43. Northern Gulf of Mexico - UNITED STATES OF AMERICA: Pine Beach: 30°,14'N/87°,44'W; Daytona Beach: 29°,12'N/80°,59'W; Ormond Beach: 29°,17'N/80°,59'W; Matanzas Outside: 29°,42'N/81°,14'W; S Ponte Vedra Beach: 30°,01'N/81°,19'W; Destin Beach: 30°,23'N/86°,29'W; Egmont Key NE: 27°,35'N/82°,45'W; Egmont Key NW: 29°,13'N/85°,24'W; Grayton Beach: 30°,19'N/86°,09'W; Honeymoon Key C: 28°,03'N/82°,49'W; Old Beach: 29°,48'N/84°,40'W; Cape San Blas SW: 29°,40'N/85°,23'W; Grand Isle C: 29°,14'N/89°,56'W; Sea Rim State Park: 29°,40'N/93°,54'W;

70. Floridian - UNITED STATES OF AMERICA: Englewood Beach: 26°,57'N/82°,23'W; Gasparilla Island BGC: 26°,44'N/82°,15'W; Gasparilla Island PM: 26°,43'N/82°,15'W; Gasparilla Island RA: 26°,44'N/82°,15'W; Pepper Beach: 27°,44'N/80°,22'W; Seagull Beach: 28°,13'N/80°,36'W; Sebastian Beach: 27°,48'N/80°,25'W

180. Southeastern Brazil - BRAZIL: Castelhanos Beach: 23°,51'S/45°,17'W; Grande Beach: 23°,51'S/45°,25'W

*Halichaetonotus marivagus* Balsamo, Todaro and Tongiorgi, 1992

Terra typica: Punta del Recisello, Isola di Capraia, Toscana, Italy: 43°,03'N/09°,48'E

Record numbers: GREECE (2), ITALY (2), BRAZIL (1); total: Wo: (4); Br: (1)

31. Aegean Sea - GREECE Akroteri White: 36°,17'N/25°,21'E; Agios Minas: 36°,21'N/27°,57'E;

35. Western Mediterranean - ITALY: Punta Ala: 42°,49’N/10°,44’E; Punta del Recisello: 43°,03’N/09°,48’E

180. Southeastern Brazil - Brazil: Grande Beach: 23°,51'S/45°,25'W

**Family** Xenotrichulidae Remane, 1927

**Genus** Heteroxenotrichula Wilke, 1954

*Heteroxenotrichula* *pygmaea* (Remane, 1934)

Terra typica: Schilksee, Germany: 54°,25'N/10°,11'E

Record numbers: FRANCE: (5); GERMANY: (5); WALES: (1); AZORE ISLANDS: (2); ITALY: (12); GREECE: (10); EGYPT: (19); UNITED STATES OF AMERICA: (29); BULGARIA: (1); BAHAMAS: (1); THE CARIBBEAN ISLANDS: (2); JAPAN: (2); GALAPAGOS (1); HAWAII: (1); BRAZIL: (2); total: Wo: (91); Br: (2)

25. North Sea - FRANCE: Andernos: 44°,43'N/01°,06'W; Trezen ar Skoden: 48°,44'N/04°,05'W; Wimereux: 50°,46'N/01°,35'E; Zuydcoote: 51°,05'N/02°,29'E; GERMANY: Ellenbogen N: 55°,03'N/08°,26'E; Litorals Station: 55°,01'N/08°,26'E; Uthörn: 55°,02'N/08°,26'E; Weststrand: 55°,02'N/08°,23'E; Schilksee: 54°,25'N/10°,11'E

26. Celtic Seas - WALES: Oxwich Beach: 51°,32'N/04°,04'W

29. Azores Canaries Madeira - AZORE ISLANDS: Porto Pim: 38°,30'N/28°,17'W; Praia do Populo: 37°,44'N/25°,36'W

30. Adriatic Sea - ITALY: Alimini: 40°,12’N/18°,26’E; San Cataldo: 40°,22’N/18°,18’E; Laguna di Grado: 45°,43’N/13°,22’E; Foce dell’Isonzo: 45°,45’N/13°,31’E; Grotto della Principessa: 39°,48’N/18°,22’E

31. Aegean Sea - GREECE: Perissa: 36°,16'N/25°,27'E; Amnissos W: 35°,19'N/25°,11'E; Kalamaki: 35°,01'N/24°,45'E; Plakias: 35°,11'N/24°,23'E; Agios Minas: 36°,21'N/27°,57'E; Ashkelon: 31°,40'N/34°,34'E; Nitzanim: 31°,44'N/34°,36'E; Palmachim N: 31°,53'N/34°,41'E; Palmachim S: 31°,53'N/34°,41'E; North Beach: 29°,32'N/34°,57'E

32. Levantine Sea - EGYPT: Betash (‘Agami): 31°,06'N/29°,43'E; Boghoush E: 31°,16'N/27°,49'E; Boghoush W: 31°,16'N/27°,49'E; Hannoville (‘Agami): 31°,06'N/29°,43'E

35. Western Mediterranean - FRANCE: Carnon: 43°,31'N/03°,57'E; ITALY: Bagnara: 41°,03’N/13°,53’E; Cuma: 40°,52’N/14°,01’E; Paestum: 40°,25’N/14°,58’E; Casamicciola: 40°,44’N/13°,54’E; Grotta del Mago: 40°,32’N/14°,04’E; Cala Liberotto: 40°,26’N/09°,50’E; Lido di Procida: 40°,44’N/14°,00’E

40. Gulf of Maine/Bay of Fundy - UNITED STATES OF AMERICA: Plum Island SW: 42°,42'N/70°,47'W

41. Virginian - UNITED STATES OF AMERICA: Roosevelt Inlet Inside: 38°,47'N/75°,09'W; Crane’s Beach: 41°,32'N/70°,41'W; Egypt Beach: 41°,45'N/70°,26'W; MBL Beach: 41°,32'N/70°,41'W; Quissett Beach: 41°,33'N/70°,40'W; Woodneck Beach: 41°,35'N/70°,39'W; Nobska Beach: 41°,31'N/70°,40'W; Matakesset Beach Inside: 41°,21'N/70°,29'W; Shinnecock Inlet I: 40°,50'N/72°,30'W

42. Carolinian - UNITED STATES OF AMERICA: Vilano Beach: 29°,55'N/81°,17'W; Bogue Inlet Beach: 34°,38'N/77°,05'W;

43. Northern Gulf of Mexico - UNITED STATES OF AMERICA: Padre Island N: 27°,28'N/97°,16'W

44. Black Sea - BULGARIA: Varna: 43°,12'N/27°,57'E

57. Oregon, Washington, Vancouver Coast and Shelf - UNITED STATES OF AMERICA: CG Beach, SL-Loc 8,9: 43°,20'N/124°,19'W; Humbug Mtn SP: 43°,21'N/124°,20'W; S Jetty O: 44°,00'N/124°,08'W; Ponsler SP: 44°,10'N/124°,07'W; CapeMears: 45°,29'N/123°,55'W; Netarts: 45°,26'N/123°,57'W; Salishan I: 44°,55'N/124°,01'W; Salishan O: 44°,55'N/124°,01'W; Seaside: 45°,59'N/123°,55'W; First Lagoon: 48°,27N/122°,59'W

63. Bahamian - BAHAMAS: East Plana Cay: 22°,37'N/73°,30'W

64. Eastern Caribbean - THE CARIBBEAN ISLANDS: Coki Bay: 18°,21'N/64°,52'W; Muller Bay: 18°,20'N/64°,51'W

70. Floridian - UNITED STATES OF AMERICA: Bahia Honda Key SE: 24°,39'N/81°,15'W; Sombrero Beach: 24°,41'N/81°,05'W; Crandon Park Inside: 25°,43'N/80°,09'W; Elliott Key Dock: 25°,26'N/80°,12'W; Pompano Beach: 26°,13'N/80°,05'W; Sebastian Beach: 27°,48'N/80°,25'W

87. Northern and Central Red Sea - EGYPT: Fâyid; 30°,19'N/32°,18'E; Beach Albatross: 27°,07'N/33°,49'E; Sharm el-Arab O: 26°,03'N/33°,54'E; Wadi `Araba: 29°,08'N/32°,39'E; Giftun IsSS: 27°,10'N/33°,57'E; Main Beach RM: 27°,44'N/34°,12'E; Moon Beach: 29°,14'N/32°,53'E; Nabq: 28°,05'N/34°,25'E; Nabq S: 28°,04'N/34°,25'E; Ras Nasrani: 27°,56'N/34°,23'E; Port Bay, Sharm: 27°,50'N/34°,14'E; Ras Sudr: 29°,36'N/32°,40'E; Tareef el-Reeh: 28°,50'N/34°,36'E; Tip RM: 27°,43'N/34°,15'E; `Uyun Musa: 29°,52'N/32°,38'E

121. South Kuroshio - JAPAN: Iriomote Jima Sta. 2: 24°,10'N/123°,53'E; Ishigaki Jima Sta. 7: 24°,09'N/124°,03'E

153. Hawaii - HAWAII: Lāʻie Beach: 21°,38'N/157°,55'W

173. Eastern Galapagos Islands - GALAPAGOS Site 5d Darwin Station: 00°,45'S/90°,17'W152.

180. Southeastern Brazil - BRAZIL: Praia do Engenho: 23°,12'S/44°,39'W; Grande Beach: 23°,51'S/45°,25'W

*Heteroxenotrichula* *squamosa* Wilke, 1954

Terra typica: Via Caracciolo, Napoli, Campania, Italy: 40°,50'N/14°, 15'E

Record numbers: DENMARK: (1); BELGIUM: (2); GERMANY: (3); SCOTLAND: (3); BRITISH ISLES: (1); WALES: (1); SCOTLAND: (10); PORTUGAL: (1); ITALY: (68); SPAIN: (1); GREECE: (19); CYPRUS: (2); EGYPT: (12); ISRAEL: (1); FRANCE: (9); UNITED STATES OF AMERICA: (26); BRAZIL: (3); total: Wo: (160); Br: (3)

25. North Sea - DENMARK: Romo: 55°,04'N/08°,33'E; BELGIUM: Mariakerke: 51°,13'N/02°,53'E; Zwin: 51°,19'N/03°,21'E; GERMANY: Dune S: 54°,10'N/07°,54'E; Litoral Station: 55°,01'N/08°,26'E; Weststrand: 55°,02'N/08°,23'E; SCOTLAND: Cruden Bay: 57°,24'N/01°,51'W; West Sands: 56°,21'N/02°,48'W; Tentsmuir Sands S: 56°,23'N/02°,48'W; BRITISH ISLES: Vazon Bay: 49°,28'N/02°,36'W.

26. Celtic Seas - WALES: Oxwich Beach: 51°,32'N/04°,04'W; SCOTLAND: Firemore Beach A: 57°,49'N/05°,41'W; Firemore Beach D: 57°,49'N/05°,41'W; Gruinard: 57°,51'N/05°,28'W; Irvine Bay A: 55°,37'N/04°,44'W; Irvine Bay C: 55°,36'N/04°,42'W; Oldshoremore: 58°,28'N/05°,08'W; East Tongue: 58°,30'N/04°,22'W;

27. South European Atlantic Shelf - PORTUGAL: Tres Irmãos: 37°,06'N/08°,33'W; FRANCE: Plage d'Eyrac: 44°,39'N/01°,10'W; Pilat Plage: 44°,36'N/01°,13'W

30. Adriatic Sea - ITALY: Alimini: 40°,12’N/18°,26’E; Grotto di Cala Corvino: 40°,58’N/17°,18’E; Torre Fortore: 41°,54’N/15°,21’E; Peschici: 41°,56’N/16°,00’E; San Cataldo: 40°,22’N/18°,18’E; Siponto: 41°,37’N/15°,54’E

31. Aegean Sea - GREECE: Akroteri Red: 36°,17'N/25°,22'E; Perissa: 36°,16'N/25°,27'E; Loutsa: 37°,56'N/24°,01'E; Varkiza: 37°,48'N/23°,48'E; Stomion: 39°,53'N/22°,51'E; Psakoudia: 40°,15'N/23°,29'E; Paralia Skotinas: 40°,02'N/22°,35'E; Plakias: 35°,11'N/24°,23'E; Amnissos E: 35°,20'N/25°,16'E; AmnissosW: 35°,19'N/25°,11'E; Georgioupolis: 35°,21'N/24°,19'E; Kalamaki: 35°,01'N/24°,45'E; Makryalos: 35°,02'N/25°,57'E; Matala: 34°,59'N/24°,45'E; Pachia Ammos: 35°,06'N/25°,48'E; Sitia: 35°,11'N/26°,06'E; Vai: 35°,14'N/26°,16'E; Zakros: 35°,05'N/26°,14'E ; Tsambika: 36°,12'N/28°,09'E

32. Levantine Sea - CYPRUS: Coral Bay: 34°,50'N/32°,21'E; Dhekelia: 34°,58'N/33°,43'E; EGYPT: Aghiba: 31°,23'N/27°,00'E; el-Arish E: 31°,08'N/33°,43'E; el-Arish W: 31°,07'N/33°,41'E; Atic Hotel: 30°,48'N/29°,03'E; Bir Mesud: 31°,14'N/29°,58'E; Boghoush W: 31°,16'N/27°,49'E; Green Beach: 31°,03'N/29°,41'E; Markaz: 30°,53'N/28°,51'E; Mersa Matruh: 31°,20'N/27°,14'E; Abd el'Rahman: 30°,59'N/28°,42'E; ISRAEL: Ashdod: 31°,48'N/34°,38'E; Netanya TN: 32°,19'N/34°,51'E; Netanya TS: 32°,19'N/34°,51'E; Palmachim N: 31°,53'N/34°,41'E; Palmachim S: 31°,53'N/34°,41'E

34. Ionian Sea - ITALY: Lido Arenella: 37°,00’N/15°,17’E; Giardini Noxos: 37°,50’N/15°,17’E; Brancaleone: 37°,58’N/16°,07’E; Marina di Ginosa: 40°,25’N/16°,49’E; Torre Lapillo: 40°,16’N/17°,51’E; Torre San Giovanni: 39°,53’N/18°,07’E; Baia Verde: 40°,00’N/18°,01’E; Lido di Policoro: 40°,11’N/16°,44’E

35. Western Mediterranean - FRANCE: Fautea: 41°,42'N/09°,24'E; Plage de Pero: 42°,08'N/08°,35'E; Marine de Pietracorbara: 42°,50'N/09°,24'E; Les Lacques: 43°,11'N/05°,41'E; Banyuls sur Mer: 42°,29'N/03°,07'E; Canet Plage: 42°,42'N/03°,01'E; St-Marie la Mer: 42°,43'N/03°,01'E; ITALY: Albisola Marina: 44°,18’N/08°,29’E; San Remo: 43°,48’N/07°,46’E; La Biodola: 42°,48’N/10°,15’E; Cavo: 42°,51’N/10°,25’E; Marina Donoratico: 43°,09’N/10°,40’E; Bagno Gorgona MP: 43°,44’N/10°,16’E; Bagni Pancaldi: 43°,32’N/10°,17’E; Marina di Pisa: 43°,44’N/10°,16’E; Seno della Perruccia: 43°,02’N/09°,47’E; Punta Braccetto: 36°,49’N/14°,28’E; Porto Palo: 37°,35’N/12°,53’E; Torre Corsari: 39°,41’N/08°,27’E; Baia di Santa Reparata: 41°,15’N/09°,09’E; Punta Ala: 42°,49’N/10°,44’E; Marina di Alberese: 42°,39’N/11°,01’E; Marina di Alberese TCL: 42°,39’N/11°,04’E; Ansedonia: 42°,24’N/11°,17’E; Castiglione: 42°,45’N/10°,51’E; Tombolo di Feniglia: 42°,24’N/11°,14’E; Tombolo Giannella: 42°,27’N/11°,09’E; Mortelliccio: 42°,56’N/10°,41’E; Bagnara: 41°,03’N/13°,53’E; Marina Camerota: 40°,00’N/15°,21’E; Via Caracciolo: 40°,50’N/14°,15’E; Cuma: 40°,52’N/14°,01’E; Lido di Fusaro: 40°,50’N/14°,01’E; Licola: 40°,54’N/14°,02’E; Paestum: 40°,25’N/14°,58’E; Palinuro: 40°,00’N/15°,15’E; Capo di Posillipo: 40°,47’N/14°,12’E; Marina di Cerveteri: 41°,59’N/12°,01’E; Fiumicino: 41°,47’N/12°,13’E; Ladispoli: 41°,57’N/12°,03’E; Anzio: 41°,31’N/12°,32’E; Montalto Marina: 42°,20’N/11°,33’E; Sabaudia: 41°,15’N/13°,02’E; Camping S. Andrea: 41°,30’N/12°,10’E; Porto S. Felice: 41°,13’N/13°,05’E; Santa Severa: 42°,02’N/11°,56’E; Torre Scissura: 41°,14’N/13°,29’E; Spiaggia di Serapo: 41°,12’N/13°,33’E; Terracina: 41°,17’N/13°,15’E; Chiaia: 40°,43’N/13°,51’E; Citara: 40°,44’N/13°,54’E; Spiaggia degli Inglesi: 40°,45’N/13°,56’E; Porto d’Ischia: 40°,45’N/13°,56’E; Jelso: 38°,27’N/14°,56’E; Jesi: 38°,22’N/14°,59’E; Cala Nave: 40°,47’N/13°,36’E; Lido di Procida: 40°,44’N/14°,00’E; San Ambrogio: 38°,02’N/14°,04’E; Trappeto: 38°,05’N/13°,03’E; San Vito lo Capo: 38°,10’N/12°,45’E; Torregaveta: 40°,47’N/14°,02’E; SPAIN: El Calô: 38°,41'N/01°,28'E

40. Gulf of Maine/Bay of Fundy - UNITED STATES OF AMERICA: Gurnet Point O: 43°,00'N/70°,40'W; Salisbury: 42°,50'N/70°,48'W; Old Orchard Beach S: 43°,29'N/70°,22'W; Seabrook Beach: 42°,53'N/70°,49'W

41. Virginian - UNITED STATES OF AMERICA: Crane’s Beach: 41°,32'N/70°,41'W; Egypt Beach: 41°,45'N/70°,26'W; MBL Beach: 41°,32'N/70°,41'W; Nauset Outside: 41°,40'N/70°,00'W; Nobska Beach W: 41°,31'N/70°,40'W; Nobska Beach M: 41°,31'N/70°,40'W; Quissett Beach: 41°,33'N/70°,40'W; Matakesset Beach Outside: 41°,21'N/70°,29'W; Matakesset Beach Inside: 41°,21'N/70°,29'W; Long Beach: 38°,27'N/76°,28'W; Indian River Inlet Outside: 38°,36'N/75°,04'W

42. Carolinian - UNITED STATES OF AMERICA: Bogue Inlet Beach: 34°,38'N/77°,05'W; North Inlet Inside: 33°,20'N/79°,10'W; North Inlet Outside: 33°,20'N/79°,10'W

43. Northern Gulf of Mexico - UNITED STATES OF AMERICA: Pensacola Beach: 30°,17'N/87°,08'W; Cape San Blas SW: 29°,40'N/85°,23'W

70. Floridian - UNITED STATES OF AMERICA: Pompano Beach: 26°,13'N/80°,05'W; Sebastian Beach: 27°,48'N/80°,25'W; Anna Marie Outside: 27°,32'N/82°,43'W; Gasparilla Island PM: 26°,43'N/82°,15'W; Mustang Island C: 27°,43'N/97°,09'W; Padre Island C: 27°,05'N/97°,23'W

87. Northern and Central Red Sea - EGYPT: Giftun IsSS: 27°,10'N/33°,57'E; Nabq: 28°,05'N/34°,25'E; ISRAEL: Coral Beach M3: 29°,30'N/34°,55'E

180. Southeastern Brazil - BRAZIL: Prumirim Beach 23º22´39.1´´S/ 44º 57´18.2´´W; Guaecá Beach 23º49´03.0´´S/45º27´51.5´´W; Castelhanos Beach 23º51´27.8´´S/45º17´20.5´´W

*Xenotrichula* *intermedia* Remane, 1934

Terra typica: Schilksee, Germany: 54°,25'N/10°,11'E

Record numbers: NORWEGIAN SEA: (2); POLAND: (7); GERMANY: (9); DENMARK: (1); BELGIUM: (3); SCOTLAND: (14); ENGLAND: (18); FRANCE: (36); WALES: (3); ISLE OF MAN: (1); IRELAND: (5); BRITHISH ISLES: (3); PORTUGAL: (1); ITALY: (53); GREECE: (13); CYPRUS: (1); EGYPT (8); ISRAEL: (5); ALGERIA: (6); UNITED STATES OF AMERICA: (53); BULGARIA: (2); ROMANIA: (3); INDIA: (1); ECUADOR: (1); BRAZIL: (9); total: Wo: (249); Br: (9)

23. Northern Norway and Finnmark - NORWEGIAN SEA: Brensholm, Kvalo I: 69°,35'N/18°,02'E; Grindø Isl. NE: 69°,37'N/18°,48'E

24. Baltic Sea - POLAND: Chalpowo: 54°,55'N/18°,21'E; Czolpino: 54°,43'N/17°,12'E; Hel Peninsula N: 54°N/18°E; Krynica Morska N: 54°,23'N/19°,28'E; Krynica Morska S: 54°,22'N/19°,28'E; Mrzezyno: 54°,15'N/15°,17'E; Wolin Park: 53°,57'N/14°,28'E

25. North Sea - GERMANY: Blidsel-Bucht: 55°,00'N/08°,23'E; Düne S: 54°,10'N/07°,54'E; Ellenbogen N: 55°,03'N/08°,26'E; Lister Tief: 55°,04'N/08°,20'E; Litoral Station: 55°,01'N/08°,26'E; Munkmarsch: 54°,54'N/08°,22'E; Puan Klent: 54°,48'N/08°,18'E; Uthörn: 55°,02'N/08°,26'E; Schilksee: 54°,25'N/10°,11'E; DENMARK: Romo: 55°,04'N/08°,33'E; BELGIUM: Bredene: 51°,14'N/02°,56'E; Oostduinkerke: 51°,06'N/02°,34'E; Zwin: 51°,19'N/03°,21'E; SCOTLAND: Collieston: 57°,20'N/01°,56'W; Cruden Bay: 57°,24'N/01°,51'W; Monifieth: 56°,28'N/02°,49'W; Rattray Head: 57°,34'N/01°,50'W; ENGLAND Seaton Carew: 54°,39'N/01°,11'W; Shotley Gate: 51°,56'N/01°,06'E; Studland: 50°,35'N/01°,56'W; Tynemouth: 55°,01'N/01°,26'W; Walton-on-the-Naze: 51°,50'N/01°,07'E; Stoup Beck: 54°,26'N/00°,32'W; Thorp Bay: 51°,30'N/00°,40'E; Whitsand Bay E: 50°,18'N/04°,14'W; FRANCE: Ambleteuse: 50°,48'N/01°,35'E; Berck-Plage: 50°,26'N/01°,33'E; Cayeux-sur-Mer: 50°,09'N/01°,29'E; Hardelot: 50°,38'N/01°,34'E; Onival: 50°,04'N/01°,23'E; Quend-Plage: 50°,20'N/01°,32'E; Wimereux: 50°,46'N/01°,35'E; Zuydcoote: 51°,05'N/02°,29'E; Aber de Roscoff: 48°,42'N/04°,00'W; Courseules: 49°,18'N/00°,28'W; Franceville: 49°,15'N/00°,12'W

26. Celtic Seas - WALES: Borth: 52°,28'N/03°,56'W; Llanbedrog: 52°,51'N/04°,34'W; Oxwich Beach: 51°,32'N/04°,04'W; ISLE OF MAN: Port Erin N: 54°,05'N/04°,46'W; IRELAND: Glenariffe: 55°,03'N/06°,02'W; Strangford Buoy: 54°,19'N/05°,28'W; Str. Lough, Entr.: 54°,20-23'N/05°,33-38'W; Keem Strand: 53°,57'N/10°,11'W; Waterville: 51°,50'N/10°,13'W; SCOTLAND: Firemore Beach A: 57°,49'N/05°,41'W; Firemore Beach D: 57°,49'N/05°,41'W; Fraserburgh: 57°,40'N/01°,59'W; Gruinard Bay: 57°,51'N/05°,28'W; Irvine Bay A: 55°,37'N/04°,44'W; Irvine Bay C: 55°,36'N/04°,42'W; Portlogan: 54°,43'N/04°,57'W; PortobelloE: 55°,56'N/03°,06'W; Portobello W: 55°,56'N/03°,06'W; Sandhead Beach: 54°,48'N/04°,56'W; ENGLAND: Bar Point: 49°,56'N/06°,18'W; Blue Anchor Bay: 51°,11'N/03°,23'W; Brancaster: 52°,58'N/00°,29'E; Exmouth Beach: 50°,35'N/03°,20'W; Hunstanton: 52°,56'N/00°,19'E; Little Bay: 49°,58'N/06°,17'W; Pakefield: 52°,27'N/01°,31'E; Par: 49°,57'N/06°,16'W; Pentle Bay: 49°,56'N/06°,19'W; Porthmellon: 49°,54'N/06°,18'W; FRANCE: Ile de Batz: 48°,44'N/04°,00'W; Ile Callot W: 48°,40'N/03°,56'W; BRITHISH ISLES: Gorey: 49°,11'N/02°,02'W; Greve du Lecq: 49°,14'N/02°,12'W; Ouaisne Bay: 49°,10'N/02°,11'W

27. South European Atlantic Shelf - FRANCE: Plage d'Angoulains: 46°,07'N/01°,10'W; Bonne Anse: 45°,41'N/01°,13'W;Chanel d'Ares: 44°,42'N/01°,12'W; Banc de Bernet: 44°,38'N/01°,14'W; Le Betey: 44°,44'N/01°,07'W; Bidart: 43°,25'N/01°,37'W; Le Canon: 44°,42'N/01°,15'W; Camp Americain: 44°,39'N/01°,15'W; Claouey: 44°,44'N/01°,13'W; Plage d'Eyrac: 44°,39'N/01°,10'W; Cap-Ferret W: 44°,37'N/01°,17'W; Gujan: 44°,39'N/01°,05'W; L'Herbe: 44°,42'N/01°,15'W; La Hume: 44°,38'N/01°,07'W; Hortense: 44°,38'N/01°,15'W; Petit Nice: 44°,33'N/01°,17'W; Le PiQuay: 44°,42'N/01°,15'W; Pyla-sur-Mer: 44°,38'N/01°,13'W; Conche St-Brice: 44°,45'N/01°,09'W; La Vigne: 44°,39'N/01°,15'W; Cassey: 44°,43'N/01°,06'W; PORTUGAL: Belliche: 37°,01'N/08°,57'W

30. Adriatic Sea - ITALY: Bibione: 45°,37’N/13°,02’E; Eraclea Mare: 45°,32’N/12°,45’E; Caorle O: 45°,35’N/12°,52’E; Francavilla al Mare: 42°,21’N/14°,17’E; Montesilvano Marina: 42°,25’N/14°,09’E; Lignano Sabbia d’Oro: 45°,40’N/13°,07’E; Pesaro: 43°,58’N/12°,55’E; Peschici: 41°,56’N/16°,00’E; Siponto: 41°,37’N/15°,54’E; Vieste: 41°,52’N/16°,10’E; Lido Porto Nuovo: 41°,51’N/16°,11’E; Petacciato Marina: 41°,11’N/14°,51’E; Ravenna Lido: 44°,28’N/12°,18’E; San Benedetto: 42°,47’N/13°,53’E; Senigallia: 43°,42’N/13°,13’E; Grotta delle Viole: 42°,06’N/15°,29’E

31. Aegean Sea - GREECE: L. Lithochorou: 40°,07'N/22°,33'E; Platamonas: 39°,56'N/22°,42'E; Loutsa: 37°,56'N/24°,01'E; Pirgadikia: 40°,18'N/23°,41'E; Salonikos: 40°,17'N/23°,40'E; Sani: 40°,05'N/23°,18'E; Tripiti: 40°,21'N/23°,53'E; Vourvourou: 40°,12'N/23°,47'E;- Stomion: 39°,53'N/22°,51'E; Amnissos W: 35°,19'N/25°,11'E; Giofiros: 35°,20'N/25°,05'E; Plakias: 35°,11'N/24°,23'E; Tsambika: 36°,12'N/28°,09'E

32. Levantine Sea - CYPRUS: Agia Napa: 34°,58'N/33°,59'E; EGYPT: Aida Hotel: 30°,50'N/29°,15'E; el-Arish W: 31°,07'N/33°,41'E; Atic Hotel: 30°,48'N/29°,03'E; Bir Mesud: 31°,14'N/29°,58'E; Cleopatra: 31°,12'N/29°,56'E; Hannoville (‘Agami): 31°,06'N/29°,43'E; Marakia Resort: 30°,54'N/29°,29'E; Abd el'Rahman: 30°,59'N/28°,42'E; ISRAEL: Ahziv: 33°,02'N/35°,05'E; Carmel TN: 32°,43'N/34°,56'E; Carmel TS: 32°,43'N/34°,56'E; Nitzanim: 31°,44'N/34°,36'E; Mikhmoret: 32°,24'N/34°,52'E

34. Ionian Sea - ITALY: San Isidoro: 40°,09’N/17°,57’E; Grotto Piccola del Ciolo 2: 39°,50’N/18°,13’E

35. Western Mediterranean - FRANCE: Canet-Plage: 42°,42'N/03°,01'E; St-Cyprien: 42°,38'N/03°,01'E; ITALY: Baratti: 43°,09’N/10°,40’E; Bagno Gorgona MP: 43°,44’N/10°,16’E; Bagni Pancaldi: 43°,32’N/10°,17’E; San Rossore: 43°,38’N/10°,15’E; Cavo: 42°,51’N/10°,25’E; Diano Marina: 43°,54’N/08°,05’E; Lerici: 44°,05’N/09°,55’E; S. Margherita C: 44°,13’N/09°,13’E; Sestri Levante: 44°,16’N/09°,23’E; Agrigento: 37°,16’N/13°,35’E; Punta Braccetto: 36°,49’N/14°,28’E; Manfria: 37°,06’N/14°,06’E; Porto Palo: 37°,35’N/12°,53’E; Lido Signorino: 37°,45’N/12°,28’E; Golfo di Dragunara: 40°,35’N/08°,11’E; Fertilia: 40°,36’N/08°,17’E; Punta Ala: 42°,49’N/10°,44’E; Castiglione: 42°,45’N/10°,51’E; Tombolo Giannella: 42°,27’N/11°,09’E; Mortelliccio: 42°,56’N/10°,41’E; Anzio: 41°,31’N/12°,32’E; Fiumicino: 41°,47’N/12°,13’E; Fregene: 41°,51’N/12°,12’E; Ladispoli: 41°,57’N/12°,03’E; Ostia: 41°,44’N/12°,16’E; Camping S. Andrea: 41°,30’N/12°,10’E; Santa Severa: 42°,02’N/11°,56’E; Spiaggia di Serapo: 41°,12’N/13°,33’E; Marina di Campo: 42°,44’N/10°,17’E; Cannelle: 42°,21’N/10°,54’E; Punta del Capel Rosso: 42°,19’N/10°,55’E;Cala Liberotto: 40°,26’N/09°,50’E; Simius: 39°,08’N/09°,33’E; Posillipo: 40°,49’N/14°,12’E; Praia a Mare: 39°,52’N/15°,46’E; ALGERIA: Les Andalouses: 35°,42'N/00°,52'W; Cap-Blanc: 35°,39'N/01°,03'W; Bomo-Plage: 35°,43'N/00°,51'W; Bouzadjar: 35°,34'N/01°,09'W; Le Chenoua: 36°,36'N/02°,26'E; St-Roch: 35°,43'N/00°,45'W

40. Gulf of Maine/Bay of Fundy - UNITED STATES OF AMERICA: Good Harbor: 42°,37'N/70°,37'W; Plum SE: 42°,42'N/70°,46'W; Plum Island S: 42°,41'N/70°,46'W; Plum Island SW: 42°,42'N/70°,47'W; Salisbury: 42°,50'N/70°,48'W; Singing Beach: 42°,34'N/70°,45'W; Gurnet Point O: 43°,00'N/70°,40'W; Saquish Neck I: 43°,00'N/70°,40'W; Situate: 42°,11'N/70°,42'W; Old Orchard Beach N: 43°,32'N/70°,20'W; Old Orchard Beach S: 43°,29'N/70°,22'W; Seabrook Beach: 42°,53'N/70°,49'W; Hampton Beach: 42°,53'N/70°,49'W

41. Virginian - UNITED STATES OF AMERICA: Indian River Inlet Inside: 38°,36'N/75°,04'W; Indian River Inlet Outside: 38°,36'N/75°,04'W; Roosevelt Inlet Inside: 38°,47'N/75°,09'W; Roosevelt Inlet Tip: 38°,47'N/75°,09'W; Gordon’s Pond: 38°,45'N/75°,04'W; Crane’s Beach: 41°,32'N/70°,41'W; MBL Beach: 41°,32'N/70°,41'W; Nauset Outside: 41°,40'N/70°,00'W; Quissett Beach: 41°,33'N/70°,40'W; Nobska: 41°,31'N/70°,40'W; Woodneck Beach: 41°,35'N/70°,39'W; Matakesset Beach Inside: 41°,21'N/70°,29'W; Breezy Point Beach: 38°,37'N/76°,30'W; Driftwood Beach: 38°,21'N/76°,23'W; Long Beach: 38°,27'N/76°,28'W; North Beach: 38°,42'N/76°,31'W; Shinnecock Inlet O: 40°,50'N/72°,30'W; Ship Bottom: 39°,38'N/74°,11'W; Buckroe Beach: 37°,02'N/76°,17'W; Community Beach: 36°,54'N/76°,07'W; First Landing, 36°,55'N/76°,03'W; Virginia B: 36°,52'N/75°,58'W

42. Carolinian - UNITED STATES OF AMERICA: Beaufort Inlet Green In: 34°,41'N/76°,40'W

43. Northern Gulf of Mexico - UNITED STATES OF AMERICA: Dauphin Island Beach: 30°,14'N/88°,09'W; Gulf State Park Beach: 30°,13'N/87°,40'W; Grayton Beach: 30°,19'N/86°,09'W ; Honeymoon Key C: 28°,03'N/82°,49'W ; Old Beach: 29°,48'N/84°,40'W; Panama City Beach: 30°,06'N/85°,46'W ; Pensacola Beach: 30°,17'N/87°,08'W; Cape San Blas SW: 29°,40'N/85°,23'W; St. George Island E: 29°,41'N/84°,44'W; St. George Island W: 29°,37'N/84°,52'W; Biloxi E: 30°,32'N/88°,55'W; Port Aransas: 27°,50'N/97°,03'W; Galveston Island: 29°,11'N/94°,57'W; Mustang Island C: 27°,43'N/97°,09'W; Padre Island S: 26°,06'N/97°,09'W

44. Black Sea - BULGARIA: Sozopol: 42°,26'N/27°,42'E; Varna: 43°,12'N/27°,57'E; ROMANIA: Agigea: 44°,03'N/28°,38'E; Constanta: 44°,10'N/28°,39'E; Mamia: 44°,15'N/28°,37'E

59. Southern California Bight - UNITED STATES OF AMERICA: Hunington Beach: 33°,41'N/118°,24'W; Long Beach: 33°,45'N/118°,08'W

107. Eastern India - INDIA: Andra Pradesh: 17°N/83°E

171. Guayaquil - ECUADOR: Los Frailes: 01°,33'S/80°,48'W

180. Southeastern Brazil - BRAZIL: Grande Beach: 23°,23'S/45°,03'W; Prumirim Island: 23°,21'S/44°,56'W; Prumirim Beach: 23°,22'S/44°,57'W; Praia do Tenório: 23°,22'S/45°,03'W; Preta e Conchas Beach: 23°,46'S/45°,42'W; Saí Beach: 23°,46'S/45°,41'W; Santiago Beach: 23°,48'S/45°,32'W; Fome Beach: 23°44'27.9"S/45°16'00.9"W; Praia da Enseada: 23°59'32.2"S/46°14'41.7"W

Genus *Draculiciteria* Hummon, 1974

*Draculiciteria tesselata* (Renaud-Mornant, 1968)

Terra typica: Forio, Isola d'Ischia, Arcipelago di Campania, Campania, Italy: 40°,44'N/13°,49'E

Record numbers: GERMANY: (3); DENMARK: (1); ENGLAND: (1); WALES: (2); ISLE OF MAN: (1); SCOTLAND (5); BRITISH ISLES: (1); IRELAND: (1); FRANCE: (6); PORTUGAL: (2); ITALY: (40); VIRGIN ISLANDS: (1); UNITED STATES OF AMERICA: (33); BRAZIL: (1); total: Wo: (97); Br: (1)

25. North Sea - GERMANY: Litoral station: 55°,01'N/08°,26'E; Munkmarsch: 54°,54'N/08°,22'E; Uthörn: 55°,02'N/08°,26'E; DENMARK: Romo: 55°,04'N/08°,33'E; ENGLAND: Brancaster: 52°,58'N/00°,29'E

26. Celtic Seas - WALES: Borth: 52°,28'N/03°,56'W; Morfa Nefyn: 52°,56'N/04°,39'W; ISLE OF MAN: Port Erin N: 54°,05'N/04°,46'W: SCOTLAND: Achnahaird: 58°,04'N/05°,18'W; Balnakiel: 58°,34'N/04°,43'W; Firemore Beach A: 57°,49'N/05°,41'W; Firemore Beach D: 57°,49'N/05°,41'W; East Tongue: 58°,30'N/04°,22'W; BRITISH ISLES: Greve du Lecq: 49°,14'N/02°,12'W; IRELAND: Keem Strand: 53°,57'N/10°,11'W

27. South European Atlantic Shelf - FRANCE: Gascony - Plage-Blan: 43°,39'N/01°,26'W; Camp Americain: 44°,39'N/01°,15'W; Claouey: 44°,44'N/01°,13'W; Le PiQuay: 44°,42'N/01°,15'W; Pyla-sur-Mer: 44°,38'N/01°,13'W; La Vigne: 44°,39'N/01°,15'W

29. Azores Canaries Madeira - PORTUGAL: Porto Formoso: 37°,49'N/25°,24'W; Praia do Norte: 38°,35'N/28°,40'W

30. Adriatic Sea - ITALY: Alberoni: 45°,21’N/12°,19’E; Cala delle Arene: 42°,07’N/15°,30’E; Bibione: 45°,37’N/13°,02’E; Eraclea Mare: 45°,32’N/12°,45’E; Torre Fortore, 41°,54'N/15°,21'E; Torre Guaceto, 40°,43’N/17°,48’E; Lignano Sabbia d’Oro: 40°,54'N/12°,57'E; Numana: 43°,25'N/13°,36'E; Lido di Porto Nuovo: 41°,51'N/16°,11'E; Pesaro: 43°,58'N/12°,55'E; Torre Vado: 39°,49'N/18°,15'E

34. Ionian Sea - ITALY: Marina di Ginosa: 40°,25’N/16°,49’E; Grotto Piccola del Ciolo 2: 39°,50’N/18°,13’E; Grotto della Principessa: 39°,48’N/18°,22’E

35. Western Mediterranean - ITALY: Cala Pisana: 35°,30’N/12°,37’E; Capo Turri: 38°,58’N/08°,26’E; Punta Ala: 42°,49’N/10°,44’E; Marina di Alberese TCL: 42°,39’N/11°,04’E, La Caletta: 40°,37’N/09°,46’E; Marina Camerota: 40°,00’N/15°,21’E; Fiumicino: 41°,47’N/12°,13’E; Forio: 40°,44'N/13°,49'E; Tombolo Giannella: 42°,27’N/11°,09’E; Cala Liberotto: 40°,26’N/09°,50’E; Chiaia di Luna:40°,54’N/12°,57’E; Montalto Marino: 42°,20'N/11°,33'E; Mortelliccio: 42°,56'N/10°,41'E; Santa Severa: 42°,02’N/11°,56’E; Spiaggia di Serapo: 41°,12'N/13°,33'E; Simius: 39°,08’N/09°,33’E; Sperlonga: 41°,16’N/13°,25’E; Tarquinia: 42°,14'N/11°,42'E; Zuccale: 42°,45'N/10°,21'E; ITA/LIG: Baratti: 43°,09’N/10°,40’E, Lido di Camaiore: 43°,55’N/10°,13’E; Punta di Civitata: 43°,01’N/09°,49’E; Genova: 44°,25’N/08°,56’E; Bagno Gorgona MP: 43°,44’N/10°,16’E; Cala della Mortola: 43°,04’N/09°,50’E; Marina di Pisa: 43°,44’N/10°,16’E

40. Gulf of Maine/ Bay of Fundy - UNITED STATES OF AMERICA: Gurnet Point Outside: 43°,00'N/70°,40'W; Plum Island S: 42°,41'N/70°,46'W; Plum Island SE: 42°,42'N/70°,46'W; Salisbury Beach: 42°,50'N/70°,48'W; Saquish Neck Inside: 43°,00'N/70°,40'W; Situate Beach: 42°,11'N/70°,42'W; NH: Hampton Beach: 42°,53'N/70°, 49'W

42. Carolinian - UNITED STATES OF AMERICA: Daytona Beach: 29°,12'N/80°,59'W; Matanzas Outside: 29°,42'N/81°,14'W; Ormond Beach: 29°,17'N/80°,59'W; S Ponte Vedra Beach: 30°,01'N/81°,19'W; St. Augustine Inlet: 29°,53'N/81°,17'W; Vilano Beach: 29°,55'N/81°,17'W; Bogue Inlet Beach: 34°,38'N/77°,05'W; Debidue: 33°,20'N/79°,10'W, North Inlet Beach Inside: 33°,17'N/79°,12'W; Beaufort Inlet Green Out: 34°,41'N/76°,40'W

43. Northern Gulf of Mexico - UNITED STATES OF AMERICA: Pine Beach: 30°,14'N/87°,44'W; Port Aransas: 27°,50'N/97°,03'W; Mustang Island C: 27°,43'N/97°,09'W; Padre Island C; 27°,05'N/97°,23'W

64. Eastern Caribbean - VIRGIN ISLANDS: St. Thomas - Coki Bay: 18°,21'N/64°,52'W

70. Floridian - UNITED STATES OF AMERICA: Anna Marie Outside: 27°,32'N/82°,43'W; Bradenton Beach: 27°,28'N/82°,42'E; Egmont Key State Park NW: 27°,45'N/82°,36'W; Englewood Beach: 26°,57'N/82°,42'W; Gasparilla Island Boca Grand Club: 26°,44'N/82°,15'W; Gasparilla Island Pass Marina: 26°,43'N/82°,15'W; Miami Beach: 25°,47'N/80°,07'W;Vero Beach: 27°,39'N/80°,21'W; Honeymoon Island: 28°,03'N/82°,49'W; Mullet Key N Outside: 27°,38'N/82°,44'W; Bahia Honda Key SE: 24°,39'N/81°,15'W; Crandon Park NW: 25°,43'N/80°,09'W

180. Southeastern Brazil - BRAZIL: Prumirim Island: 23°,21'S/44°,56'W

**Order** Macrodasyida Remane, 1925 [Rao and Clausen, 1970]

**Family** Planodasyidae Rao & Clausen, 1970

**Genus** *Crasiella* Clausen, 1968

*Crasiella fonseci* Hochberg, 2014

Terra typica: Tamanduá Island, Caraguatatuba, São Paulo, Brazil: 23°35'24.0"S/45°16'48.0"W

Record numbers: BRAZIL (2); total: Wo: (0); Br: (2)

180. Southeastern Brazil - BRAZIL: Tamanduá Island: 23°35'24.0"S/45°16'48.0"W; Fome Beach: 23°44'27.9"S/45°16'00.9"W

**Family** Dactylopodolidae Strand, 1929

**Genus** *Dactylopodola* Strand, 1929

*Dactylopodola baltica* (Remane, 1926)

Terra typica: Kieler Bucht, Germany: 54°N/10°E

Record numbers: POLAND: (1); GERMANY; (10); DENMARK: (2); SCOTLAND: (12); FRANCE: (7); SWEDEN (1); WALES: (3); IRELAND: (2); ISLE OF MAN (1); ENGLAND: (6); GREECE: (1); EGYPT: (2); UNITED STATES OF AMERICA: (9); BRAZIL: (1); total: Wo: (57); Br: (1)

24. Baltic Sea - POLAND: Hel Peninsula N/S: 54°N/18°E

25. North Sea - GERMANY; Boknis Eck: 54°,31'N/10°,01'E; Buhne List-Sued: 55°,00'N/08°,25'E; Düne N/NW: 54°,11'N/07°,53'E; Ellenbogen S: 55°,02'N/08°,27'E; Litoral Station: 55°,01'N/08°,26'E; Flüggesand: 54°,25'N/10°,58'E; Kieler Bucht: 54°N/10°E; Kieler Förde: 54°,46'N/10°,14'E; Strander Bucht: 54°,25'N/10°,11'E; Weissenhaus: 54°,18'N/10°,46'E; DENMARK: Langeland-Belte: 54°,48'N/10°,52'E; Vejsnaes-Flach: 54°,44'N/10°,27'E; SCOTLAND: Cruden Bay: 57°,24'N/01°,51'W; FRANCE: Courseules: 49°,18'N/00°,28'W; Franceville: 49°,15'N/00°,12'W; Villers-sur-Mer: 49°,17'N/00°,00'W; SWEDEN: Fiskebackskil: 58°,20'N/11°,30'E

26. Celtic Seas - WALES: Black Rocks: 53°,17'N/04°,07'W; Llanfairfechan: 53°,14'N/04°,03'W; Traeth Bychan: 53°,20'N/04°,16'W; ISLE OF MAN: Derby Haven: 54°,05'N/04°,39'W; IRELAND: Ballymaconell: 54°,40'N/05°,38'W; Strangford Harbor: 54°,22'N/05°,32'W; SCOTLAND: Achiltibuie: 58°,00'N/05°,23'W; Gruinard Bay: 57°,51'N/05°,28'W; Kirkcolm: 54°,58'N/05°,04'W; Laggan: 56°,51'N/05°,49'W; Peanmeanach: 6°,51'N/05°,46'W; Portobello E: 55°,56'N/03°,06'W; Portobello W: 55°,56'N/03°,06'W; Samalaman Island: 56°,49'N/05°,49'W; Sandhead Beach: 54°,48'N/04°,56'W; Sandwood Bay: 58°,32'N/05°,06'W; Tentsmuir Sands S: 56°,23'N/02°,48'W; FRANCE: Ile de Batz: 48°,44'N/04°,00'W; Banc de Duslen/Pighet: 48°,43'N/03°,58'W; Plouneour-Trez: 48°,40'N/04°,18'W; Aber de Roscoff: 48°,42'N/04°,00'W; ENGLAND: Appletree Flats: 49°,56'N/06°,20'W; Bar Point: 49°,56'N/06°,18'W; Brancaster: 52°,58'N/00°,29'E; Hunstanton: 52°,56'N/00°,19'E; Old Quay Flats: 49°,57'N/06°,17'W; Pentle Bay: 49°,56'N/06°,19'W

31. Aegean Sea - GREECE: Neo Kalikratia: 40°,17'N/23°,05'E

32. Levantine Sea - EGYPT: Bir Mesud: 31°,14'N/29°,58'E; Cleopatra: 31°,12'N/29°,56'E

40. Gulf of Maine/Bay of Fundy - UNITED STATES OF AMERICA: Plum Island S: 42°,41'N/70°,46'W; Salisbury: 42°,50'N/70°,48'W; Seabrook Beach: 42°,53'N/70°,49'W

41. Virginian - UNITED STATES OF AMERICA: Sussex - Roosevelt Inlet Tip: 38°,47'N/75°,09'W

42. Carolinian - UNITED STATES OF AMERICA: Cape Hatteras Sta.6: 33°,35'N/76°,39'W; Cape Hatteras Sta.7: 33°,50'N/76°,39'W; North Inlet Inside: 33°,20'N/79°,10'W

70. Floridian - UNITED STATES OF AMERICA: Pompano Beach: 26°,13'N/80°,05'W; Crandon Park N: 25°,43'N/80°,09'W

180. Southeastern Brazil - BRAZIL: Castelhanos Beach: 23°,51'S/45°,17'W

*Dactylopodola todaroi* Garraffoni, Di Domenico & Hochberg, 2017

Terra typica: Fome Beach, IlhaBela Island, São Paulo, Brazil: 23°44'27.9"S/45°16'00.9"W

Record numbers: BRAZIL (1); total: Wo: (0); Br: (1);

180. Southeastern Brazil - BRAZIL: Fome Beach: 23°44'27.9"/S/45°16'00.9"W

**Genus** *Dendrodasys* Wilke, 1954

*Dendrodasys* aff. *rubomarinus* Hummon, 2011

Terra typica: Fome Beach, IlhaBela Island, São Paulo, Brazil: 23°44'27.9"S/45°16'00.9"W

Record numbers: BRAZIL (1); total: Wo: (0); Br: (1)

180. Southeastern Brazil - BRAZIL: Fome Beach: 23°44'27.9"S/45°16'00.9"W

**Family** Macrodasyidae Remane, 1926

**Genus** *Macrodasys* Remane, 1924

*Macrodasys fornerise* Todaro & Rocha, 2004

Terra typica: Segredo Beach: São Sebastião, São Paulo, Brazil: 23°,49'S/45°,25'W

Record numbers: BRAZIL (2); total: Wo: (0); Br: (2)

180. Southeastern Brazil - BRAZIL: Segredo Beach: 23°,49'S/45°,25'W; 23°37'50.0"S/45°23'06.3"W

**Genus** *Urodasys* Remane, 1926

*Urodasys viviparus* Wilke, 1954

Terra typica: Castel dell’Ovo, Napoli, Campania, Italy: 40°,15'N/14°,16'E

Record numbers: GREECE: (11); CYPRUS: (2); ITALY: (21); FRANCE: (1); BERMUDA: (1); PANAMA: (1); UNITED STATES OF AMERICA: (10); EGYPT: (11); ISRAEL: (3); INDIA: (4); BRAZIL: (2); total: Wo: (65); Br: (2)

30. Adriatic Sea - ITALY: Porto Colombo: 41°,05’N/17°,05’E; Cala Paura: 41°,00’N/17°,13’E; Miramare: 45°,41’N/13°,43’E; Grotta del Sale: 42°,06’N/15°,29’E; Cala Spino: 42°,07’N/15°,30’E; Grotta delle Viole: 42°,06’N/15°,29’E

31. Aegean Sea - GREECE: Neo Fokea: 37°,43'N/23°,55'E; Loutsa: 37°,56'N/24°,01'E; Varkiza: 37°,48'N/23°,48'E; Neo Kalikratia: 40°,17'N/23°,05'E; Kehres: 37°,55'N/23°,01'E; Fourni: 36°,05'N/27°,45'E; Glystra: 36°,02'N/28°,00'E; Lindos: 36°,04'N/28°,06'E; Ierissos: 40°,22'N/23°,52'E; Khrisi: 34°,52'N/25°,45'E; Makryalos: 35°,02'N/25°,57'E

32. Levantine Sea - CYPRUS: Governor's Beach: 34°,42'N/33°,16'E; Yeroskipos: 34°,43'N/32°,25'E

34. Ionian Sea - ITALY: S.M. di Leuca: 39°,47’N/18°,18’E

35. Western Mediterranean - FRANCE: - Lagune du Brusc: 43°,03'N/05°,47'E; ITALY: Torre al Bagno: 43°,02’N/09°,50’E; Genova; 44°,25’N/08°,56’E ; Bagno Gorgona MP: 43°,44’N/10°,16’E; Marina di Pisa; 43°,44’N/10°,16’E;- Golfo di Dragunara: 40°,35’N/08°,11’E; Punta Ala: 42°,49’N/10°,44’E; Bagnetielli: 40°,44’N/13°,55’E; Casamicciola: 40°,44’N/13°,54’E; Porto d’Ischia: 40°,45’N/13°,56’E; Via Caracciolo: 40°,50’N/14°,15’E; Castel dell’Ovo: 40°,50’N/14°,16’E; Palinuro: 40°,00’N/15°,15’E; Cala Nave: 40°,47’N/13°,36’E; Zuccale: 42°,45’N/10°,21’E

62. Bermuda - BERMUDA: 32°N/64°W

67. Southwestern Caribbean - PANAMA: Galeta Island: 09°,24'N/79°,53'W

70. Floridian - UNITED STATES OF AMERICA: Mullet Key NI: 27°,38'N/82°,43'W; Adams Key: 25°,24'N/80°,14'W; Crandon Park NW: 25°,43'N/80°,09'W; Crandon Park N: 25°,43'N/80°,09'W; Crandon Park Inside: 25°,43'N/80°,09'W; Crandon Park Outside: 25°,43'N/80°,09'W; Elliott Key Dock: 25°,26'N/80°,12'W; Sands Key: 25°,30'N/80°,11'W; Monroe - Bahia Honda Key SE: 24°,39'N/81°,15'W; Big Pine Key STB: 24°,39'N/81°,20'W

87. Northern and Central Red Sea - EGYPT: M Bareika N: 27°,46'N/34°,12'E; Giftun VSI: 27°,10'N/33°,49'E; Main Beach RM: 27°,44'N/34°,12'E; Middle Garden: 27°,54'N/34°,21'E; Moon Valley: 27°,15'N/33°,48'E; Mugawish: 27°,08'N/33°,49'E; Na'ama Bay S: 27°,53'N/34°,19'E; Nabq S: 28°,04'N/34°,25'E; Princess: 27°,09'N/33°,49'E; Safaga: 25°,44'N/33°,57'E; Sharm el-Sheikh: 27°,50'N/34°,15'E; ISRAEL: Coral Beach M4: 29°,30'N/34°,55'E; Coral Beach N1: 29°,30'N/34°,55'E; Snuba Dive Shop: 29°,29'N/34°,54'E

105. Maldives - INDIA: Agatti Island: 10°,51'N/72°,11'E; Adda Atoll: 00°,40'S/73°,07'E

107. Eastern India - INDIA: Lawson’s Bay: 17°,44'N/83°,20'E

109. Andaman and Nicobar Islands - INDIA: Mayabinder: 12°,44'N/92°,57'E

180. Southeastern Brazil - BRAZIL: Prumirim Island: 23°,21'S/44°,56'W; Vermelha Beach: 23°,11'S/44°,38'W

**Genus** *Kryptodasys* Todaro, Dal Zotto,Kånneby & Hochberg, 2019

*Kryptodasys* *carlosrochai* Todaro, Dal Zotto,Kånneby & Hochberg, 2019

Terra typica: São Paulo State, Ilhabela Island - Castelhanos Beach: 23°51'27.8"S/45°17'20.5"W

Record numbers: BRAZIL (1); total: Wo: (0); Br: (2)

180. Southeastern Brazil - BRAZIL: Castelhanos Beach: 23°51'27.8"S/45°17'20.5"W

**Family** Thaumastodermatidae Remane, 1929

**Genus** *Pseudostomella* Swedmark, 1956

*Pseudostomella dolichopoda* Todaro, 2012

Terra typica: Grande Beach, Ubatuba, São Paulo, Brazil: 23°23'04.4"S/45°03'49.9"W

Record numbers: BRAZIL (12); total: Wo: (0); Br: (12)

76. Eastern Brazil - BRAZIL: 20°02'24.0"S/40°10'48.0"W

180. Southeastern Brazil - BRAZIL: Grande Beach 23°23'04.4"S/45°03'49.9"W; Prumirim Island: 23°21'55.1"S/44°56'30.2"W; Prumirim Beach: 23°22'39.1"S/44°57'18.2"W; Praia do Tenorio: 23°22'57.5"S/45°03'41.1"W; Grande Beach: 23°23'04.4"S/45°03'49.9"W; Guaecá Beach: 23°49'03.0"S/45°27'51.5"W; Santiago Beach: 23°48'37.1"S/45°32'26.9"W; Saí Beach: 23°46'25.7"S/45°41'44.2"W; Preta e Conchas Beach: 23°46'16.8"S/45°42'49.8"W; Castelhanos Beach: 23°51'27.8"S/45°17'20.5"W; Vermelha Beach: 23°25'05.4"S/45°02'20.4"W

*Pseudostomella squamalongispinosa* Araujo, Balsamo & Garraffoni, 2014

Terra typica: Segredo Beach: São Sebastião, São Paulo, Brazil: 23°,49'S/45°,25'W

Record numbers: BRAZIL (3); total: Wo: (0); Br: (3)

76. Eastern Brazil - BRAZIL: Pontal da Barra Beach: 17º53' S/39º21' W; Lugar Comum Beach: 17º54' S''/39º21' W; Pau Fincado Beach: 17º54' S'/39º22' W.

**Genus** *Ptychostomella* Remane, 1926

*Ptychostomella lamelliphora* Todaro, 2013

Terra typica: Grande Beach, IlhaBela Island, São Paulo, Brazil: 23°51'S/45°25'W

Record numbers: BRAZIL (2); total: Wo: (0); Br: (2)

180. Southeastern Brazil - BRAZIL: Grande Beach 23°51'S/45°25'W; Fome Beach: 23°44'27.9"S/45°16'00.9"W
